# A facile alternative strategy of upcycling mixed plastic waste into vitrimers

**DOI:** 10.1038/s42004-023-00949-8

**Published:** 2023-07-27

**Authors:** Kok Wei Joseph Ng, Jacob Song Kiat Lim, Nupur Gupta, Bing Xue Dong, Chun-Po Hu, Jingdan Hu, Xiao Matthew Hu

**Affiliations:** 1 School of Material Science and Engineering, Nanyang Technological University, Nanyang Avenue, 639798 Singapore, Singapore; 2 Temasek Laboratories, Nanyang Technological University, 50 Nanyang Drive, 637553 Singapore, Singapore; 3 Nanyang Environment and Water Research Institute, Nanyang Technological University, 637141 Singapore, Singapore; 4 Rolls-Royce@NTU Corporate Lab, Nanyang Technological University, 50 Nanyang Avenue, 639798 Singapore, Singapore

**Keywords:** Polymers, Composites, Organic molecules in materials science, Mechanical properties, Sustainability

## Abstract

Chemical depolymerization has been identified as a promising approach towards recycling of plastic waste. However, complete depolymerization may be energy intensive with complications in purification. In this work, we have demonstrated upcycling of mixed plastic waste comprising a mixture of polyester, polyamide, and polyurethane through a reprocessable vitrimer of the depolymerized oligomers. Using poly(ethylene terephthalate) (PET) as a model polymer, we first demonstrated partial controlled depolymerization, using glycerol as a cleaving agent, to obtain branched PET oligomers. Recovered PET (RPET) oligomer was then used as a feedstock to produce a crosslinked yet reprocessable vitrimer (vRPET) despite having a wide molecular weight distribution using a solventless melt processing approach. Crosslinking and dynamic interactions were observed through rheology and dynamic mechanical analysis (DMA). Tensile mechanical studies showed no noticeable decrease in mechanical strength over multiple repeated melt processing cycles. Consequently, we have clearly demonstrated the applicability of the above method to upcycle mixed plastic wastes into vitrimers and reprocessable composites. This work also afforded insights into a potentially viable alternative route for utilization of depolymerized plastic/mixed plastic waste into crosslinked vitrimer resins manifesting excellent mechanical strength, while remaining reprocessable/ recyclable for cyclical lifetime use.

## Abstract (web summary)

Chemical depolymerization is a promising approach to recycle plastic waste, but complete depolymerization is energy-intense. Here, the authors show upcycling of mixed plastic waste to highly-crosslinked, reprocessable vitrimers through incomplete depolymerization using glycerol as a cleaving agent.

## Introduction

The high durability^1^ of commodity plastics has contributed to their unsustainable accumulation and pervasiveness in our environment. According to reports, 95% of plastic packaging materials, worth about 80–120 billion USD annually^1^, are lost to the economy only after their first use. Furthermore, in 2015 alone, ~302 million metric tons of plastic^2^ waste was generated, with packaging accounting for 141 million metric tons^2^. However, to manage the exponential increase in plastic waste, conventional approaches such as burning^3^ or disposing of them at landfills^4^ have grown increasingly unsustainable^3,4^. To ameliorate these approaches, methods for recycling waste plastics have been developed which mainly fall into two categories, mechanical and chemical recycling.

Mechanical recycling^5^ is by far the most common approach. Generally, plastic types are often mechanically broken down into small flakes^5,6^ which are then melted, processed, and made into new products. This technique is, however, susceptible to contaminants^7–9^ which would require extensive sorting and/or washing of the waste material, incurring additional cost. Consequently, mechanical recycling of multilayer packaging would be far less effective. Furthermore, mechanical recycling can lead to a decrease in recyclate quality, resulting in downcycling^10^. This limits the number of reuse cycles while only prolonging the life of the material.

Chemical recycling may be more effective for extensively contaminated or mixed plastic systems. Traditional chemical recycling aims to obtain monomeric building blocks^11^ through depolymerization. Through this process, it is possible to convert end-of-life products into monomers^7,12,13^ while facilitating separation from impurities^7^. This idealized approach seeks to create a closed-looped economy with little to no waste. Plastic wastes which are virtually impossible to recycle mechanically, such as multilayer packaging^8^ and thermosets^14^, could be recycled using such techniques. However, problems from chemical recycling persist. Different plastics exhibit different reactivities and may chemically interact with miscellaneous components, such as additives, complicating^7^ depolymerization. Multiple reactive species can cause various side reactions to occur^7,15^, hampering target molecule separation and purification.

Thus, recycling mixed plastics systems remains a key challenge for traditional recycling. Repolymerization of a mixed plastic feedstock can lead to immiscibility and incompatibility between polymer types^16–18^. As a result, rigorous sorting and cleaning of mixed plastic waste is still required^18^, generally lowering the cost-effectiveness of plastic recycling. Beyond standard recycling methods, increasing efforts to upcycle plastic waste are gaining prominence in the field of sustainability^13^. Apart from traditional recycling, the upcycling objective is not always to reclaim the original polymer or material, but rather to repurpose it for novel applications^13,19–23^ providing more opportunities to convert plastic waste into more valuable products. One popular strategy includes the chemical  deconstruction of polymeric material to form oligomeric^15,19,21^, instead of monomeric, feedstocks. This is especially suitable for common condensation type polar polymers^13^, such as polyethylene terephthalate (PET), polyamide, and polyurethane (PUR), where the main chain either undergoes partial hydrolysis^22^ or bond exchange reactions^24–26^ to cause scission of the main chain. This can result in polyols with reactive end groups suitable for functionalization for further application. Hence, it can potentially reduce the need for extensive purification^19,22^ and creates opportunities for the application of other reagents (i.e. catalysts, crosslinkers, or additives) for functionalization and chemical modification. An example can be observed in a recent study, from Kimura and Hayashi^22^, demonstrating a “one-shot transformation^22^” of polyester, with the addition of an epoxy crosslinker, directly creating a crosslinked polyol vitrimer. Where the polyester main chain was degraded into oligomers and its end groups were functionalized with an epoxy species to afford a crosslinked, yet reprocessable polymeric material. Upcycling allows for greater flexibility in chemical modifications, developing additional possible applications for the reintegration of plastic waste. This can also strengthen and supplement conventional recycling processes by overcoming some limitations seen in chemical and mechanical recycling. A more direct approach to upcycling plastic waste would be to attain new polymeric materials. Studies have shown the relation of the molecular weight of the material matrix to its toughness^27–29^ thus, an intuitive method would be the incorporation of crosslinkers to increase it and enhance the strength of the material^6^. However, this consequently reduces its reprocessability which merely delays its end-of-life. Thus, to retain reprocessability and contribute to a circular economy^30^, the explicit formation of dynamic cross-linkages in recyclates can lead to vitrimers from plastic waste, as seen from work done by Kimura and Hayashi^22^.

Vitrimers are reprocessable, but crosslinked, polymers due to the presence of crosslinked covalent adaptive networks^31,32^ (CANs), allowing covalent bonds to dynamically rearrange in response to external stimuli. This is attributed to reversible exchange reactions, such as (non-exhaustively) transesterification, transcarbamoylation^33^, and transamination^31^ to afford dynamic covalent bonds. The concept underlying vitrimers, vitrimerisation, can also be applied to crosslinked systems while preserving recyclability for a circular economy. Sheppard et al.^34^ demonstrated the application of carbamate exchange on post-consumer polyurethane, upcycling it to a crosslinked yet recyclable material. Further studies include Zhou et al.^35^, who successfully degraded and repolymerize poly(butylene terephthalate) (PBT) via transesterification reactions with glycerol, and Qiu et al.^36^, introducing hyperbranching into PET bottles through transesterification with polyol amine and reinforcement with epoxy via reactive extrusion. Both works by Zhou et al. and Qiu et al. capitalized on the branched nature of polyols to afford crosslinked polymers but relied on transesterification exchanges to maintain recyclability and a closed-loop economy. However, some PET bottles were extruded^36^ with epoxy as a chain-extender to supplement crosslinking compensating for molecular weight reduction. Albeit at a small amount, crosslinking can, conceptually, be introduced through grafting and integration of the polyol which was demonstrated by Qiu et al., as well as Zhou et al. with no further crosslinkers. Unfortunately, the work done with PBT/Glycerol did not report on mechanical strength for benchmarking the vitrimer which may warrant further exploration. Fundamentally, current studies are mostly restrained on clean and/or virgin plastic subjects while mixed plastic systems remain an enduring problem.

Nonetheless, existing studies^22,34–36^ suggest that vitrimerisation can be a viable alternative to upcycle post-consumer plastics. Instead of complete depolymerization, controlled depolymerization to oligomers may be less resource intensive and oligomers still possess reactive end groups which can be functionalized for crosslinking. Formation of CANs would enable recyclability and contribute to a closed-loop economy but may also homogenize different plastic species, with comparable chemistry and reactivity, increasing compatibility. This may be of advantage for very contaminated or multilayer plastic systems.

**Fig. 1:**
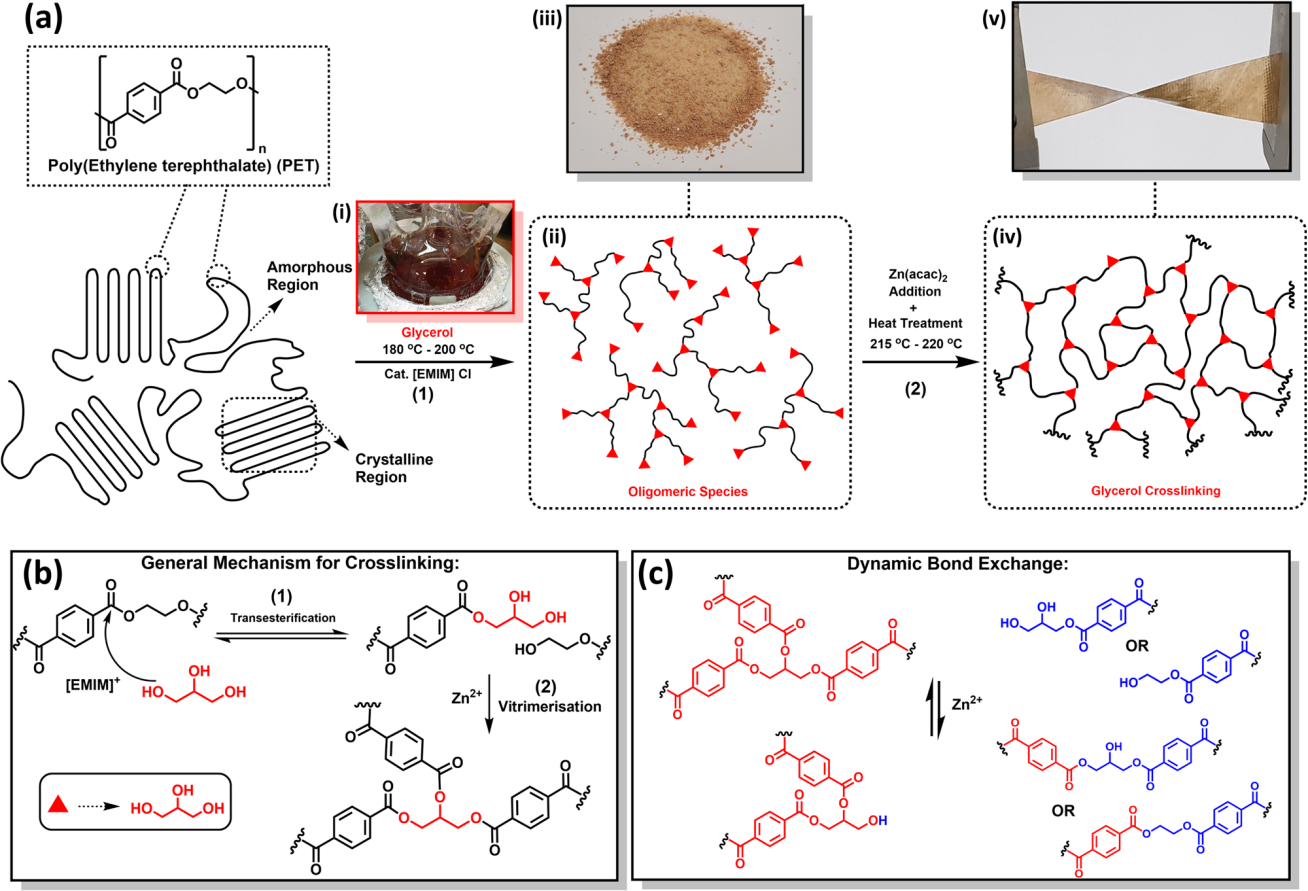
Experimental concept towards depolymerization and vitrimerisation of PET. Schematic illustration of concept and reaction scheme for PET depolymerization and crosslinking with glycerol before applications on mixed plastic systems. **a** Experimental scheme where (1) PET chain scission by glycerol in the presence of the catalytic amount of [EMIM] Cl, in NMP as a solvent, to form a dark red reaction mixture (i), to produce glycerol grafted polyol oligomers (ii) which are precipitated (iii) and isolated as Recovered PET (RPET). (2) Through the addition of zinc catalyst followed by heat treatment, crosslinked network (iv) is formed from RPET and can be processed into a film (v). **b** Scheme showing (1) Carbonyl exchange (transesterification) with glycerol degrades the bulk PET into branched oligomeric PET. (2) Catalytic addition of a Zn-containing species into RPET encourages glycerol insertion which contributes to the crosslinking of the material. **c** Mechanism for dynamic bond exchanges via further transesterification for processibility after crosslinking.

To realize this concept, this work employs a model polymer, PET, to recognize the process of vitrimerisation for upcycling which can subsequently be applied to mixed plastic systems. The experimental scheme and upcycling strategy for this study are depicted in Fig. 1. PET is depolymerized under controlled conditions in n-methyl-2-pyrrolidone (NMP) solvent to produce oligomeric moieties. Glycerol is used as a dual-functional cleaving and crosslinking reagent for vitrimerisation in the PET model study. Similar to reactions with EG to attain BHET^37^, glycerol causes scission of the main chain, via transesterification, followed by glycerol being grafted on the end groups of PET oligomers^38^. Glycerol subunits then serve as crosslinkers eliminating the need for additional crosslinkers to significantly increase molecular weight. A higher amount of polyol was added compared to similar existing studies^35,36^ to achieve more glycerol-grafted moieties and to obtain oligomers of lower molecular weight. Furthermore, smaller molecular weight oligomers easily dissolve in NMP enabling the separation of any immiscible impurities from the reaction mixture, which may be beneficial when working with a mixed plastic system. Oligomeric species can easily be precipitated in water, and filtered, to obtain the oligomeric Recovered PET (RPET) feedstock. This simplifies and reduces the required purification steps to achieve a viable feedstock. After cross-linking, existing carbonyl groups can participate in transesterification exchanges with polyols resulting in CANs, facilitated by a zinc catalyst, allowing the vitrimer to be reprocessable, recyclable, and circular in the application.

Conceptually, this process is not limited to PET but is also applicable to other polar polymers^13^, such as polyamides and polyurethanes, which can undergo glycolysis and similar bond exchange reactions. This gives rise to the possibility of its application to mixed plastic systems consisting of similar polymer materials. Hence, to quantify and determine the efficacy of the proposed method for applying vitrimerisation as a viable process for depolymerization, repolymerization, and reprocessability, PET was used as a model polymer first. Subsequently, this method would be applied to a simulated composition of real mixed plastic waste to demonstrate vitrimerisation as a viable approach towards upcycling. Ultimately, PET was successfully converted into an oligomeric feedstock that can intrinsically crosslink. The produced crosslinked oligomers demonstrated relatively strong mechanical properties whilst maintaining good reprocessability, thus achieving vitrimers. The overarching concept was demonstrated through a waste-mixed plastic system yielding oligomeric material which can also be utilized as a viable feedstock. To further demonstrate the versatility of the process towards application, waste mixed plastic oligomers were upcycled as a feedstock, via a continuous process, to fabricate a reprocessable composite.

## Results and discussion

Perpetuating from a previous study^19^, branched PET oligomers were obtained after controlled partial depolymerization of PET. To assess the effects of a higher amount of glycerol on PET depolymerization, repolymerization, and material fabrication (upcycling), three feedstocks were synthesized through controlled polymer depolymerization, by the reagent amount presented in Table 1, to obtain the oligomeric RPET feedstocks. The ratios given in Table 1 refer to the number of ethylene terephthalate repeating units to the theoretical hydroxyl content of the oligomeric samples, varied 5:1, 5:3, and 1:1, which shall be referred to as RPET 5:1, RPET 5:3, and RPET 1:1. For example, RPET 1:1 signified for every one ethylene terephthalate repeating unit, there is one hydroxyl group. The hydroxyl content is provided by the amount of glycerol added to the system which will correspond to the ratio given for PET depolymerization.

**Table 1:** Reagent table for controlled depolymerization of PET.

Starting reagents			Depolymerized oligomeric feedstock					
			Feedstock 1		Feedstock 2		Feedstock 3	
Name	Structure	Function	RPET 5:1		RPET 5:3		RPET 1:1	
			(g)	(wt%)	(g)	(wt%)	(g)	(wt%)
Poly(ethylene terephthalate) (PET)		Model polymer chain	200	96.0	200	90.4	200	85.5
Glycerol		Cleaving agent and crosslinker	6.39	3.1	19.17	8.7	31.94	13.7
1-Ethyl-3-methylimidazolium chloride ([EMIM] Cl)		Polymer depolymerization catalyst	2	1.0	2	0.9	2	0.9

Reagent table for PET depolymerization to yield three oligomeric feedstocks RPET 5:1, RPET 5:3, and RPET 1:1. For each variation, the glycerol amount is the ratio of the number of ethylene terephthalate repeating units ([ET]_*n*_) to the number of alcohol groups (–OH) content, given by glycerol, to achieve the oligomeric RPET feedstocks. The ratio is used as a label for each variation as RPET [ET]_*n*_ : (–OH). The mass of each reagent, followed by its overall weight content, is given in the table for each variation. Note: NMP was used as a solvent for depolymerization in a 1:1 weight ratio (200 g) with respect to PET.

Glycerol was utilized as the cleaving agent, with NMP as solvent, under the catalytic influence of ionic liquid, 1-Ethyl-3-methylimidazolium chloride ([EMIM] Cl). Ionic liquids were utilized because they have been shown to be excellent transesterification catalysts^24,39,40^. Imidazolinium-based ionic liquids have been shown to be capable of interacting with Lewis bases to form N-heterocyclic carbenes^41^ with significantly enhanced reactivity to promote transesterification. Furthermore, the ionic liquid can be removed, by washing with water, allowing better catalytic control with the zinc catalyst for RPET formulation subsequently. NMP solvent was reduced and optimized to a weight ratio of 1:1 to PET. Relatively little solvent is required for this process when compared to other processes, such as solvent extraction of polymers by physical dissolution^42^.

### PET depolymerization and RPET analysis

**Fig. 2:**
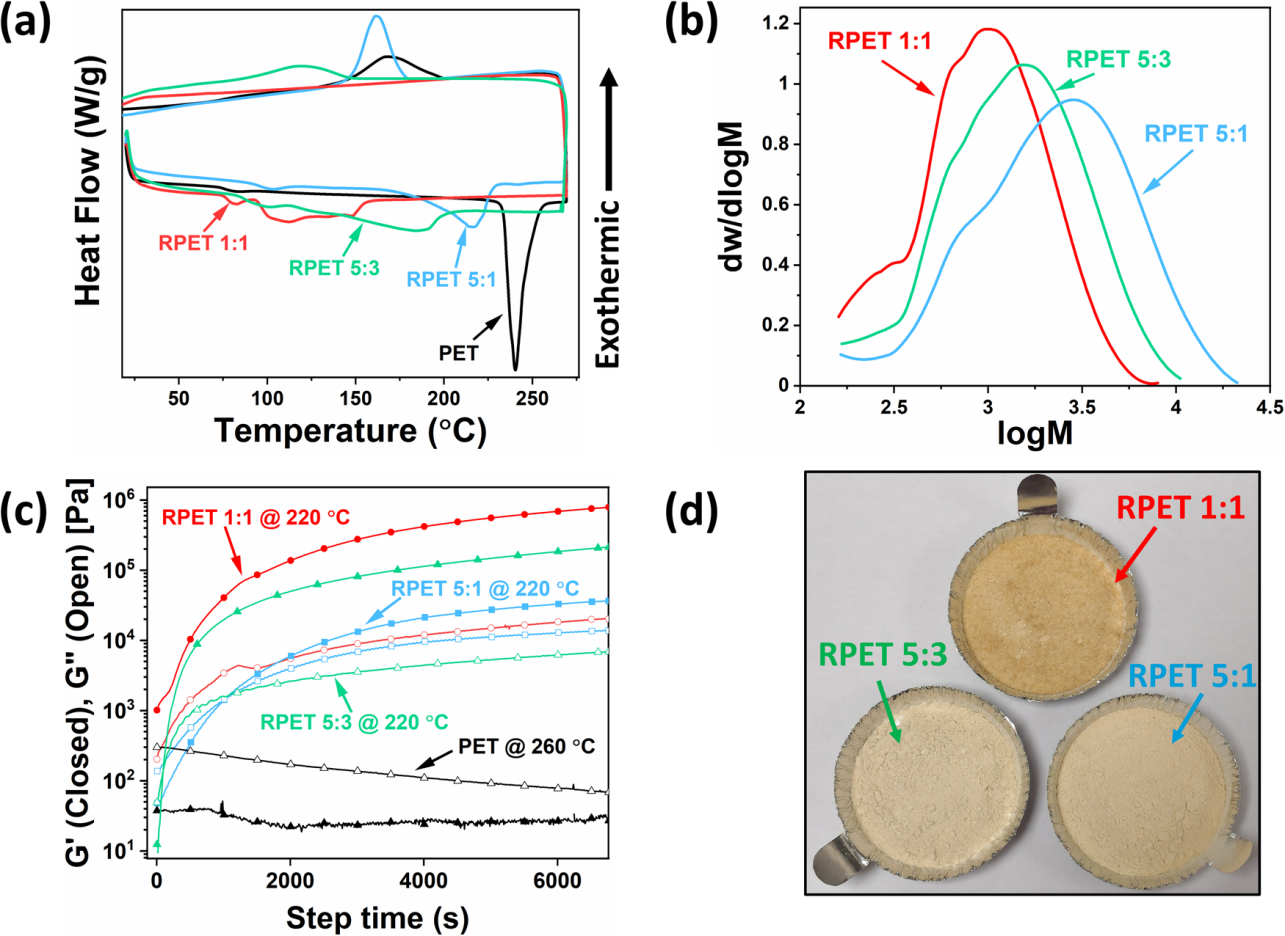
Oligomeric RPET Characterization and Analysis. Characterization of RPET feedstocks obtained from controlled depolymerization of PET with glycerol of varying amounts. **a** Differential scanning calorimetry temperature sweep of RPET compared to neat PET under a heating and cooling rate of 10 °C/min. Exothermic upwards. **b** Molecular weight distribution of RPET samples after gel permeation chromatography. The system was calibrated relative to polystyrene from 162 Da to 45.12 kDa. **c** Rheology results obtained for an isothermal oscillation at 1 Hz and 1% strain for 2 h. For all RPET samples, *G*’ (closed symbols) and *G*” (open symbols) changes were observed at 220 °C while for neat PET, *G*’ and *G*” changes were observed at its melting point of 260 °C. **d** Obtained grinded RPET 5:1, 5:3, and 1:1 powder feedstock.

Based on a previous study^19^, glycolysis was driven to equilibrium after 18 h at 180 °C^19^. PET oligomers were then recovered through precipitation in water to serve as the Recovered PET (RPET) feedstock. Slight discoloration of the RPET feedstocks was observed which could be attributed to the imidazolium-based ionic liquid used which degraded^43^ during the reaction temperature. This could lead to the discoloration observed in vitrimer fabrication subsequently. Characterizations of RPET feedstocks are within expectations where observed trends correspond to the amount of glycerol introduced during glycolysis (Fig. 2).

DSC analysis of RPET yielded lower and broader melting endotherms when compared to the sharp endotherm at 240 °C for neat PET (see Fig. 2a). This directly results from transesterification reactions of PET with glycerol which yielded PET oligomers of varying molecular weights. During controlled cooling, exotherms can be observed for RPET 5:1, RPET 5:3, and neat PET indicating crystallization of longer linear polymer chains. Through size exclusion chromatography, the *M*_n_ and *M*_w_ of PET were found to be 32.06 kDa and 54.80 kDa, respectively (Table S1). GPC analysis further corroborates the presence of oligomeric species with a wide molecular weight distribution for RPETs (Fig. S1 and Table S1). With the addition of more glycerol, the degree of depolymerization is more substantial, with RPET 1:1 having smaller molecular weights when compared to RPET 5:3 and 5:1.

However, according to DSC, the recrystallization exotherms disappeared for RPET 1:1 during the cooling cycle. Higher amounts of glycerol would result in more significant chain scission resulting in smaller chain oligomers with trifunctional glycerin subunits^19^. With shorter chains, the number of reactive hydroxyl groups per unit volume, provided by glycerol, would be significantly higher leading to faster repolymerization kinetics and crosslinking. This is likely to have taken place during the heating cycle, resulting in a crosslinked sample that prevents recrystallization during the cooling cycle (see Fig. 2a). The crosslinking kinetics, influenced by glycerol amount, were investigated through the isothermal rheology of RPET. Rheology was done on melt samples of RPET, mixed with a catalytic amount (5 mol%) of zinc acetylacetonate (Zn(acac)_2_) and compared to neat PET. RPET samples were subjected to isothermal oscillation at 220 °C while PET was subjected to its melting temperature, of 260 °C for two hours. PET behaved as a typical melt polymer where its loss modulus (*G*”) is higher than its storage modulus (*G*’) and where no crosslinking behavior and reaction is seen and expected (see Fig. 2c). However, for the RPET samples, the reversal of G’ and G” is observed. RPET 5:1 initially behaved as a polymer melt, but a crossover gel point was observed after 1056 s, approximately 17 min. This implies that the material exhibits a higher degree of elasticity, transitioning away from liquid-like behavior and entailing crosslinking^44–46^. RPET 5:3 and RPET 1:1 exhibited faster crosslinking and relatively higher *G*’ values when compared to RPET 5:1. According to the rubber elastic theory, the storage modulus, *G*’, is linearly related^45^ to the crosslink density ρ_x_ through the equation, *G*’ = *ρ*_*x*_*RT*, where *R* and *T* are the gas constant and temperature respectively^45^. This suggests that RPET 5:3 and 1:1 possesses higher crosslink densities, when compared to RPET 5:1, likely due to their higher glycerol contents.

The crosslinking, and chain recombination, mechanism is conceptually a transesterification-driven polycondensation^35^ between oligomeric end groups with the elimination of small molecules, this is further elaborated with fundamental kinetics in the Supplementary Information (Supplementary Note 1), where glycerol replaces ethylene glycol subunits resulting in trifunctional cross-linkages. With higher amounts of glycerol used for PET glycolysis, higher amounts of glycerol subunits would be grafted on PET oligomers. Thus, the hydroxyl concentration of the system would dramatically increase, speeding up transesterification exchanges which would facilitate condensation of ethylene glycol, glycerol insertion and, consequently, crosslinking. DSC and rheology results corroborate the proposed kinetics mechanism of crosslinking influenced by glycerol content. Furthermore, higher amounts of glycerol would also result in more glycerolic cross-linkages which would translate into higher crosslink density.

### vRPET vitrimer analysis

RPET feedstocks were compounded with catalytic amounts Zn(acac)_2_, 5 mol% with respect to amount of PET repeating units, according to Table S2, and processed to produce RPET vitrimers (vRPET). Zn(acac)_2_ is a common catalyst for transesterification and its larger organic counter ion may better facilitate its miscibility in the organic polymeric system. It would be the preferred catalyst of choice for impelling dynamic bond exchanges within CANs.

### Mechanical characterization of vRPET

**Fig. 3:**
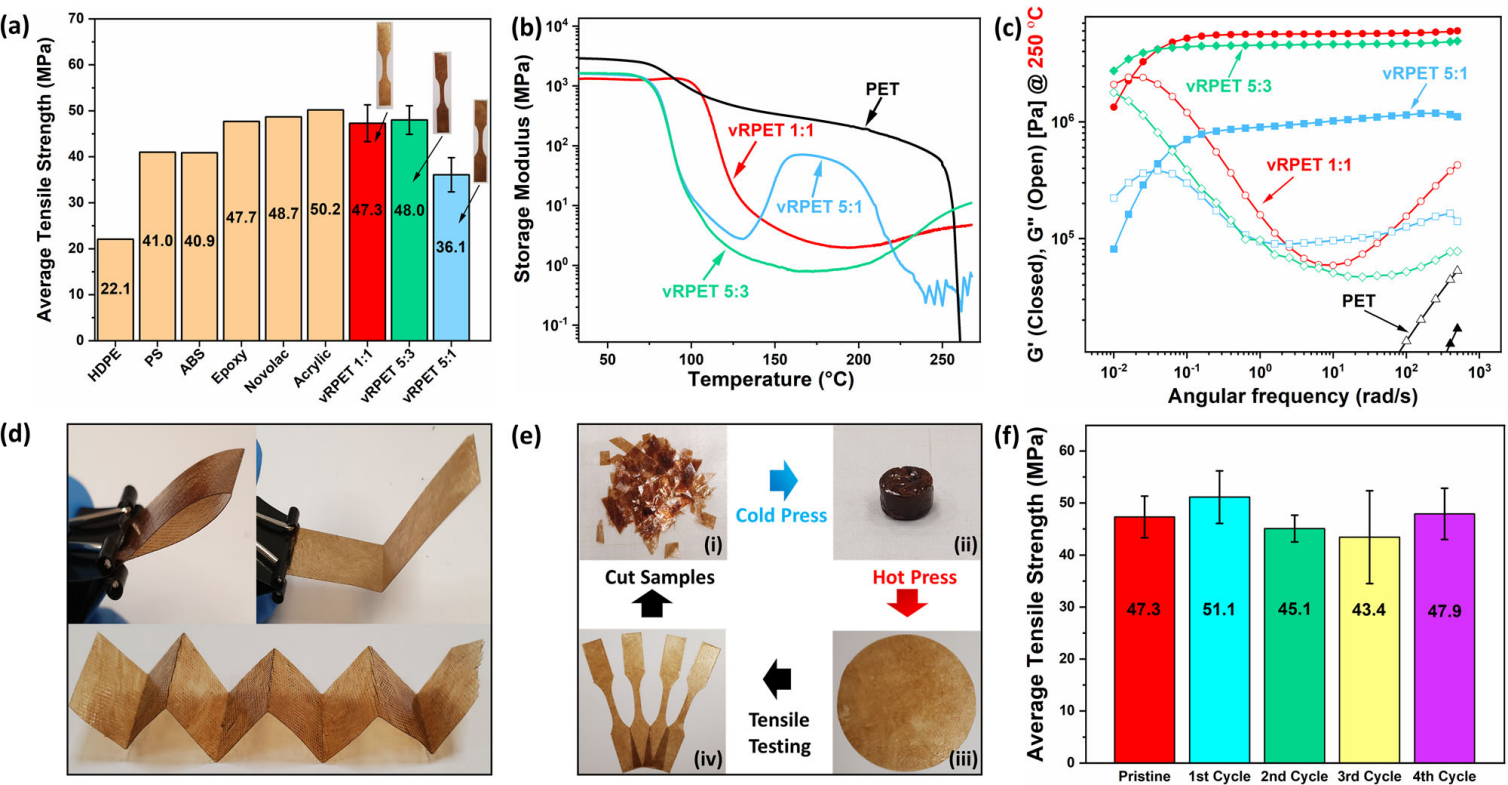
RPET vitrimers (vRPET) characterization and reprocessability assessment. Analysis and characterization of fabricated crosslinked vitrimers, vRPET. **a** Comparison of the average ultimate tensile strength of vRPET films obtained from RPET 1:1, RPET 5:3, and RPET 5:1 with error bars, error bars were produced due to sample variations during tensile testing of vRPET films, to other common commercial polymers: High-density polyethylene (HDPE)^61^, polystyrene (PS)^62^, acrylonitrile butadiene styrene (ABS)^63^, epoxy^64^, phenolic resins (Novolac)^65^ and acrylic^66^ (average values taken from online database, Matweb, at time of writing). **b** Dynamic mechanical analysis (DMA) curves of vRPET films and neat PET at a heating rate 3 °C/min. **c** Small angle oscillation rheology, on samples of vRPET 1:1, vRPET 5:3, vRPET 5:1 and neat PET, from an angular frequency range of 500–0.01 rad/s at an isothermal temperature of 250 °C. **d** Folded samples of vRPET 1:1 film. Films fabricated exhibited relatively good toughness,  which resisted breaking  when hard fold(s) were applied. **e** Recycling process of vRPET 1:1 film. (i) Samples were cut into small  pieces and (ii) cold pressed into a pellet of 10 mm diameter. (iii) Pellet then hot pressed to produce a reprocessed film which was (iv) cut into dogbone samples for tensile testing. After tensile testing, broken and leftover samples are then cut into smaller pieces for the repetition of the cycle. **f** Ultimate tensile strength, with error bars, of vRPET 1:1 after 4 cycles of recycling shows little change in mechanical strength. Error bars were produced due to sample variations during tensile testing of vRPET 1:1 films after each cycle of recycling.

Error bars, seen in Fig. 3a, f, and Table 2, are due to sample variations during tensile mechanical testing of vRPET films. Tensile film testing showed that the vRPET samples (Fig. S6) performed well, with no additional additives or crosslinkers added other than the catalytic amount of Zn(acac)_2_, and their average tensile strength, elongation, and Young’s modulus are summarized in Table 2. Benchmarking vRPET films, films with higher glycerol content, vRPET 1:1 and vRPET 5:3, demonstrated good tensile strength when compared to some common commercial thermoplastics and thermosets (see Fig. 3a), such as epoxy and polystyrene, implying them as potential alternatives for some common virgin plastics. The resulting vRPET films possessed comparable strength to Qiu et al. work, on upcycling PET with an amine polyol and epoxy^36^, and higher strength than the polyester vitrimer fabricated by Kimura and Hayashi^22^.

**Table 2:** Physical properties of vRPET films.

Sample	*T*_g_ (°C)	Average tensile strength (MPa)	Ultimate elongation (%)	Young’s modulus (GPa)
vRPET 1:1	106.4	47.3 ± 4.0	5.3 ± 0.5	1.06 ± 0.11
vRPET 5:3	80.4	48.0 ± 3.1	4.4 ± 0.5	1.27 ± 0.06
vRPET 5:1	81.6	36.1 ± 3.7	4.7 ± 0.8	1.16 ± 0.09

Summary of physical properties of RPET vitrimers (vRPET) film samples for this study.

### Influence of glycerol on vRPET

Dynamic mechanical analysis (DMA) (Fig. 3b) showed PET behaving as a typical semicrystalline polyester, melting at temperatures above 250 °C, approximately at the PET melting point. However, vRPET films did not melt at temperatures above 250 °C (Figs. 3b and S3) but rather exhibited a rubbery plateau resulting from their cross-linked networks^35^. A noticeable curve was observed for vRPET 5:1 and vRPET 5:3, which can be attributed to further crosslinking reactions^47^. Two heating cycles of DMA were performed on all vRPET films (Fig. S4), resulting in a significant reduction of the amplitude of curves initially observed. This implies that the rate of crosslinking for vRPET5:1 and vRPET 5:3 is slower than that of vRPET 1:1. As previously discussed, a reduced glycerol content is expected to result in a lower hydroxyl concentration, which kinetically slows the rate of crosslinking. The glass transition (*T*_g_) temperature of the samples provides further evidence for the different extent of cross-linking. Given by the loss modulus maxima (Fig. S5) and summarized in Table 2, vRPET 5:1 and vRPET 5:3 exhibited very similar *T*_g_ to virgin PET however, vRPET 1:1 had a *T*_g_ that was 23.7 °C higher. This suggests vRPET 1:1 possessed a more rigid structure due to a prominent crosslinked network due to its larger glycerol content, requiring more energy to induce its glassy transition state, resulting in a higher *T*_g_.

### CANs and dynamic behavior of vRPET

The dynamic nature and rheological behavior were studied through small angle oscillation (SAO) on thermally cured vRPET samples and compared to virgin PET at high temperatures (see Fig. 3c). Samples were subjected to isothermal frequency sweeps and tested at low strain amplitudes (1%) to probe only the linear viscoelastic properties of the samples.

For virgin PET at 240 °C, its elastic modulus (*G*’) is situated above its loss modulus (*G*”) curve (Fig. S9). However, at higher temperatures, a significant decrease in virgin PET’s *G*’ was observed with its *G*” being higher than its *G*’ (Fig. S10). This suggests that PET has melted at 250 °C indicating more liquid-like behavior, which is the expected behavior for the thermoplastic. In contrast, vRPET samples generally exhibited a higher *G*’ than *G*” for both mentioned temperatures. This is further observed in SAO of vRPET 1:1 at temperatures above the melting point of PET at 250, 260, and 270 °C. (Figure S11). This implies vRPET samples behave more elastically despite being subjected to high temperatures signifying their resistance to melting. This strongly suggests the presence of a crosslinked network^45^ that does not allow the material to melt completely. Hence, vRPET samples have transited away from PET thermoplastic behavior to a crosslinked thermoset.

A crossover point was observed for vRPET samples (Fig. 3c) at longer time scales, where the *G*’ and *G*” exhibited similar frequency scaling, which corresponds to the gel-point conversion (i.e., *G*” ~ *G*’ ~ *ω*^Δ^)^44,45^ in accordance with the Winter–Chambon criterion. The presence of the crossover point, despite the samples’ crosslinked nature, implied the presence of CANs^45^ which allows the material to exhibit liquid-like stress relaxation. Additionally, CANs present in vRPET are fundamentally transesterification exchanges, categorized as reversible exchange rearrangement^45^, which does not disassociate but rather only relax via bond rearrangement^45^ and is reflected at longer time scales where the sample is allowed to undergo viscoelastic relaxation^45^.

It is also observed, in Fig. 3c, that the vRPET samples possess different crossover points at different timescales. vRPET 5:1 has the crossover point at the shortest time scale, followed by vRPET 1:1 then vRPET 5:3, which is lower than the observable frequency of the equipment. A plausible explanation could be due to vRPET 5:1 longer molecular chain which allows better chain mobility. Coupled with its lower crosslink density, as seen from its *G*’, allows for more thermoplastic behavior, which relaxes more easily compared to the other two samples. vRPET 5:3 possesses a higher crosslink density which lowers chain mobility. However, it still possesses reactive esters and hydroxyl groups which allow it to undergo dynamic bond exchanges to facilitate relaxation, albeit at longer timescales. vRPET 1:1 also possesses high crosslink density and undergoes relaxation at shorter timescales than vRPET 5:3. This could be due to higher amounts of reactive groups, due to its highest glycerol content, which allows for more efficient dynamic bond exchanges causing easier relaxation, and as such, a crossover point of shorter timescale.

### Recovery and recyclability of vRPET 1:1

On the basis that more reactive groups would cause better bond exchanges, and relatively better mechanical performance, vRPET 1:1 was chosen for recovery and recyclability experiments. Moreover, vRPET 1:1 films fabricated were tougher and more resistant to breaking when corrugated or distorted (Fig. 3d). Possessing a higher crosslink density translates to higher molecular weight which imparts toughness. Further evidence for significant crosslinking is corroborated through an XRD comparison of PET and the vRPET 1:1 (Fig. S13). The diffractogram obtained from PET shows the expected characteristics of crystallinity relative to planes (010), (010), (110), and (100) for scattering angles 2*θ* = 16.4°, 18°, 23.3°, and 26.5°, respectively^48^. However, the diffractogram of vRPET 1:1, for this study, described a completely amorphous polymer structure. This random arrangement was achieved by vRPET 1:1 crosslinked morphology, significantly reducing crystallinity^49^, implying substantial crosslinking. This can be analogous to Fig. 1a, depicting an amorphous crosslinked arrangement by the trifunctional glycerol.

Despite being significantly crosslinked, vRPET 1:1 still maintains good reprocessability. This is demonstrated by performing four cycles of recycling, where the process described in Fig. 3e, followed by a comparison of its mechanical strength after each cycle. As vRPET 1:1 was relatively tough and grinding it into powder proved difficult, the film was cut into smaller pieces and pelletized via cold pressing, ensuring that all parts of the film were subjected to the recycling process achieving better homogeneity. The pelletized sample was then hot-pressed to produce a recycled film which was then cut into dog-bone samples for tensile testing. vRPET 1:1 film was recycled four times via the same process which showed little change in tensile strength (Figs. 3f and S7). This suggests vRPET 1:1 has ample reactive groups to undergo efficient dynamic covalent bond exchanges allowing its CANs to achieve near-full recovery after each recycling cycle. This suggests good reprocessability which can allow vRPET 1:1 to be (re)molded to a variety of shapes. Herein, as an example, vRPET 1:1 was molded into a button-like disk (Fig. S2).

### Application of vitrimerisation towards mixed plastic upcycling

Through the utilization of glycerol, which also can be a waste product^50^, it is possible to easily achieve polyol oligomers to serve as a viable feedstock for vitrimer fabrication. Through the studies done with PET above, it is possible to achieve a relatively strong and highly crosslinked, yet reprocessable, vitrimer. Conceptually, the process with glycerol is not limited to PET but step-growth polymers^13^ susceptible to glycolysis, should be able to be processed and upcycled in a similar fashion. This will be demonstrated in the following section of this study involving mixed plastics from real waste.

**Fig. 4:**
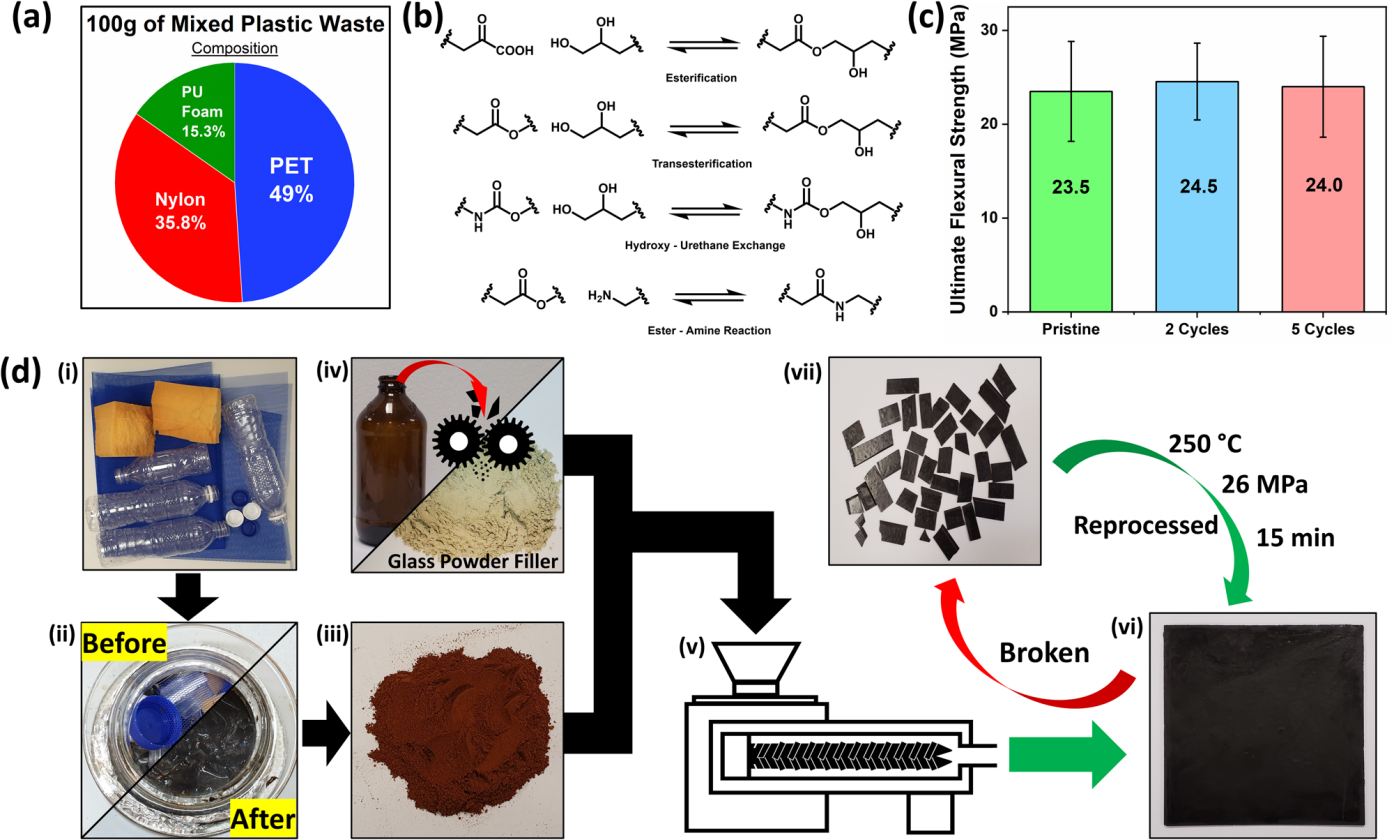
Application of vitrimerisation to upcycle mixed plastics. Demonstration of the potential feasibility of this work, on mixed plastic waste. **a** Composition of simulated mixed plastic waste for this demonstration, based on an existing study^2^ on waste composition. **b** A series of chemical reactions that may occur to facilitate crosslinking and vitrimerisation. **c** Flexure strength of composite after 2 cycles and 5 cycles of reprocessing compared to the pristine sample. Error bars were produced due to sample variations during the 3-point flexural bend. **d** Scheme for the plastic waste upcycling process. (i) Actual plastic waste for depolymerization was shredded and placed into a reaction vessel. (ii) Plastic waste placed in a reaction vessel (Before) was solubilized in solvent at 180 °C forming a dark slurry (After) after 20 min. (iii) A red precipitate was achieved which was washed and collected as recovered mixed plastic (RMP). (iv) A waste glass beverage bottle was ground into powder particles, with a diameter of approximately 150 microns. (v) 80 wt% of waste glass powder was extruded with RMP at 220 °C at 80 rpm. (vi) The extruded sample was hot pressed to afford a 12 cm × 12 cm square tile. (vii) The resultant tile is reprocessable. The tile was broken apart and can be reformed when hot-pressed at 250 °C and 20 MPa for 15 min.

As mentioned in earlier sections, the objective of this study is not the fabrication of new materials but to investigate vitrimerisation as an alternative approach to upcycling mixed plastic waste. To demonstrate the efficacy of utilizing glycerol towards vitrimerisation and upcycling mixed plastics, a composition of waste plastic types, based on data collected by Geyer et al.^2^, was gathered. Geyer et al. reported that in 2015, PET, PUR, and polyester, polyamide, and acrylic (PP&A)^2^ generated 32, 16, and 42 million metric tons of waste respectively^2^. For this work and simplicity, PP&A would be deemed wholly a polyamide. The mass ratio adopted for this study would be approximately based on the reported data, which is indicative and suggestive that the composition proposed in this study would be reflective of some common plastic waste observed globally. The mass ratios of the mixed waste plastic used in this study are shown in Fig. 4a. The rationale for the selection of plastic types, for this study, would be the chemical similarities between esters, amides, and urethanes. These functional groups share similar molecular structures where they all contain an electrophilic carbonyl carbon susceptible to nucleophilic attack. Glycolysis exploits this electrophilicity to enable chain scission. The glycolysis of polyesters^40^, polyamides^25,51^, and polyurethane^26,52^ are known areas that resulted in molecular moieties grafted with the polyol used^25,26,40,51,52^. Amalgamating the concepts of glycolysis with the processes presented in this work, it is possible to attain polyester, polyamide, and polyurethane oligomers grafted with glycerol. Furthermore, all mentioned species are likely to undergo glycolysis in a one-pot reaction to produce mixed plastic oligomers with end groups being predominantly hydroxy groups, thereby recovering some degree of control. Reactive polymer types would solubilize to facilitate separation from non-reactive species and impurities. For this demonstration, polyolefin bottle caps and labels were included in the one-pot depolymerization which would represent unreactive species in the reaction. Consequently, the formation of a polyolefin layer was observed on the surface after 18 h, which can be removed. With the removal and separation of unreactive species from the reaction mixture, depolymerized mixed plastic polyols can be then isolated for upcycling.

### Recovered mixed plastics (RMP) as a viable feedstock

The process of acquiring mixed plastic oligomeric polyols is similar to the process presented for RPET using glycerol, [EMIM] Cl, and NMP as solvent. Plastic composition described in Fig. 4a was liquified after 18 h and was precipitated and washed with water to yield a dark red powder that would be referred to as recovered mixed plastic (RMP). DSC on RMP shows multiple broad melting endotherms as low as 75 °C (Fig. S15a) which suggests the initial waste plastics have been successfully depolymerized. The FTIR spectra (Fig. S14) of the sample show a strong broad band at 3300 cm^−1^, which is indicative of an O–H stretch, as expected of a polyol sample. Data provided by the spectra shows a sharp peak at 1721 cm^−1^ showing C = O stretch for ester. The additional peak at 3297 cm^−1^ can be assigned to –NH bending vibrations^53^ and 1641 and 1546 cm^−1^ may be attributed to –NC = O bending vibrations^53^ may be contributed by polyamide and polyurethane oligomer polyols species. As the RMP is obtained from actual mixed plastic waste, it is difficult to accurately characterize every functional group, but it does indicate the presence of multiple functional groups that can undergo repolymerization and vitrimerisation.

RMP is an oligomeric mixture that possesses numerous functional groups to allow multiple reactions to occur to enable repolymerization and crosslinking. Figure 4b illustrates some of the possible reactions which may occur during vitrimerisation. Esterification reactions can occur with carboxylic (COOH) end-groups from polyamides oligomers^25^, transesterification reactions are expected between polyols and ester groups, and hydroxy–urethane exchanges^54^ can occur as well. Furthermore, reactions between amine end groups, from polyamides^51^, and esters may occur, albeit less favorable and less in occurrence. These series of chemical bond exchanges may insert glycerol into the polymer network facilitating repolymerization and crosslinking to afford RMP vitrimer (vRMP). Evidence of crosslinking can be observed via isothermal rheology, at 220 °C of RMP mixed with the same catalytic weight percentage of zinc, from Zn(acac)_2_, as the formulation for vRPET. It was observed that the RMP mixture melted at 116 °C, where its *G*’ fell below its *G*” (Fig. S12). The sample behaves like a polymer melt, exhibiting relatively low viscosities, until temperatures around 165 °C where a gel point was observed. At 220 °C, the sample exhibited *G*’ values above its *G*” signifying crosslinking of the oligomeric mixture. This corroborates with the results obtained from the rheology of RPET. Moreover, the end groups of RMP oligomers should be predominantly glycerol hydroxyl subunits that can undergo covalent bond exchanges. This minimizes, if not prevents, phase separation between mixed plastic species during repolymerization to achieve a greater degree of homogeneity. DSC analysis on a vRMP film (Fig. S15b) fails to show sharp melting and recrystallizing endotherms and exotherms corresponding to different plastic species. This suggests vRMP to be crosslinked and relatively homogenous with no obvious endotherms corresponding to individual plastic components, akin to a typical random copolymer^55^.

### vRMP glass composite

To further demonstrate the versatility of using vitrimerisation as an upcycling process would be the fabrication of a healable and reprocessable vRMP composite. Since RMP is oligomeric initially, it possesses lower molecular weights which translates to lower melt viscosities. This creates the opportunity to fabricate composites with high filler content. A common filler to utilize would be silica, which is a common mineral found in stone, concrete, and, most of all, glass. Glass, particularly colored glass, has been a growing waste stream^56^ that is difficult to recycle. Hence, to both enhance the material properties and upcycle waste glass, an empty glass bottle was utilized as the silica source and a filler for the vRMP composite.

To develop a continuous process and exploit oligomeric RMP low melt viscosity, RMP was extruded with 80 wt% glass powder. The resultant product was then hot pressed into a stiff 12 cm by 12 cm square tile, of ~1 mm thickness, then thermally cured. The tile was then cut into smaller rectangle tiles and subjected to a three-point bending test. The flexural modulus and strength of the vRMP tile were found to be 9.83 ± 2.86 GPa and 23.5 ± 5.3 MPa, respectively, which is comparable to limestone^57^ and commercial construction bricks^58^. Thermogravimetric analysis of the extruded composite further confirms at least 80% weight of silica (Fig. S8) present in the sample. Additionally, observation of the cross-section of the extruded vRMP composite tile via scanning electron microscope (SEM) (Fig. S16) shows little to no voids between the glass particles and vRMP polymer, indicating good adhesion between the binder and the filler. This is likely due to RMP’s high degree of hydrogen bonding, which promotes interaction and adhesion between the polyol binder and the glass filler^59^. To demonstrate circularity, recyclability experiments were performed by subjecting the vRMP composite through 5 cycles of reprocessing. vRMP composite shows little change in flexure strength (Figs. 4c and S17), comparable to vRPET 1:1, suggesting excellent recoverability and circularity, for a material made almost entirely from waste.

## Conclusion

In this study, it has been demonstrated that vitrimerisation is a viable upcycling alternative. Through solvent-assisted glycolysis, PET was depolymerized under controlled conditions to yield polyol oligomers. Following this, the polyol oligomers were directly repolymerized and upcycled in a solventless process, without the need for additional crosslinkers. Even oligomers with a wide molecular weight distribution can be used directly in the fabrication of vitrimers, which is an added benefit of the process. Consequently, the use of this upcycling method eliminates one of the limitations of the conventional recycling method, namely the need for extensive purification of the recyclates.

It was further shown that the process is not limited to PET but is also applicable to other condensation polymers, such as polyamide and polyurethane. Experiments using mixed plastic waste not only suggest this but also demonstrate the robustness of the process to handle multiple plastic species with minimal cleaning. The process can also facilitate the separation of oligomers from impurities and immiscible plastics, during the controlled depolymerization step, which can be segregated by either phase separation or simple filtration. This can be particularly useful for upcycling multilayer packaging or heavily mixed plastic systems, supplementing where conventional mechanical and chemical recycling may fall short. Oligomeric polyols can then be recovered through precipitation in water, which can be subsequently repolymerized to form crosslinked yet reprocessable vitrimers. Through covalent exchange interactions, different plastic species can be homogenized into a co-polymer network. This recovers and creates value from  waste mixed plastic systems, that would otherwise be too complex to recycle.

Furthermore, the process allows for flexibility to create opportunities for innovation using waste plastic oligomeric feedstocks for various applications, such as composite creation (using waste glass) and maybe ionogels^19^. Application demonstrations on actual waste collected from waste disposal points may suggest efficacy for waste originating from existing landfills or environments. Further investigations into waste plastics found in the mentioned locations, where plastics are heavily contaminated and weathered, could be useful in determining the viability of this concept.

## Methods

### Materials and reagent

1-methyl-2-pyrrolidinone (>99% Purity) and chloroform (HPLC/ Spectroscopic, Amylene Stabilized) was purchased from Tedia. Bis(2,4-pentanedionato) Zinc(II) (>96% purity) was purchased from TCI. 1-Ethyl-3-methylimidazolium chloride (>95%), Glycerol (99.5% purity), and 1,1,1,3,3,3-Hexafluoro-2-propanol (>99%) was purchased from Sigma Aldrich. Food-grade poly(ethylene terephthalate-co-isophthalate) copolymer (Ramapet N1) was supplied by Indorama Ventures. All reagents and solvents were used without further purification. PET bottles and polyurethane foam was collected at various waste disposal points while nylon netting was purchased from a local textile supplier. A colored glass bottle was collected after a beverage, purchased from a local supermarket, was consumed.

### Depolymerization towards Recovered PET (RPET)

200 g of PET was dissolved in 200 g of NMP at 180 °C to obtain a dark orange reaction mixture. A measured amount of glycerol and [EMIM] Cl was then added to the reaction mixture in accordance with Table 1 for controlled depolymerization. A near-instantaneous decrease in viscosity was observed. The reaction mixture was heated at 180 °C for 18 h, then quenched in deionized water (500 mL) at ambient temperature, followed by precipitation. Precipitate was removed via vacuum filtration and placed into 500 mL of deionized water with stirring for washing. This cycle was repeated twice. Precipitate was then collected through vacuum filtration and dried in a convection oven at 80 °C to obtain recovered PET (RPET) compositions, yields of polymeric material recovery were calculated against initial mass of PET (200 g): RPET 5:1 (192 g, yield of recovery: 96%), RPET 5:3 (193 g, yield of recovery: 97%), and RPET 1:1 (170 g, yield of recovery: 85%).

### vRPET 5:1 and vRPET 5:3 film fabrication

RPET 5:1/ RPET 5:3 and Zn(acac)_2_ were grinded via pestle and mortar separately and then dry mixed in accordance with the mass formulation in Table S2. The powder mixture was then added to a Thermo Scientific HAAKE MiniLab II twin screw extruder and cycled internally for 2 h at 220 °C at 80 rpm. The prepolymer mixture was then extruded and further heat treated at 220 °C for 2 h. The resultant product was then cold pressed into pellets (0.5–1.0 g) of 10 mm diameter at ~7 tonnage. Pellet was then hot-pressed at 25 MPa at 250 °C for 30 min to obtain films of vRPET 5:1/ vRPET 5:3.

### vRPET 1:1 film fabrication

RPET 1:1 and Zn(acac)2 were ground via pestle and mortar separately and then dry mixed in accordance with the mass formulation in Table S2. The powder mixture was then added to a 50 mL round bottom flask and placed in an oil bath at 215 °C. Powder mixture formed a low viscosity melt and was left at 215 °C with stirring for 2 h. The pre-polymer melt was then poured out onto a Teflon film adhered to a steel plate and then heat treated for a further 2 h at 220 °C. The resultant product was then cold pressed into pellets (0.5–1.0 g) of 10 mm diameter at ~7 tonnage. Pellet was then hot-pressed at 25 MPa at 250 °C for 30 min to afford films of vRPET 1:1.

### RPET 1:1 reprocessability experiments

1.5 g of vRPET 1:1, after initial tensile testing on a pristine sample, was cut into smaller pieces and cold pressed at 7 tons into a pellet of 10 mm diameter. Pellets were then hot-pressed at 250 °C for 30 min to afford a recycled vRPET 1:1 film. The film was then cut into dog-bone-shaped specimens to produce a uniform profile of samples with a long central of (*l* × *w* × *t*) 10 mm × 3 mm × ~0.085 mm for tensile testing. Residual samples and specimens after tensile testing were then subjected to the above-mentioned procedure to afford recycled film for another cycle of tensile testing. This cycle was performed four times.

### Depolymerization of mixed plastics to afford recovered waste mix plastic (RMP)

PET plastic bottles were collected from disposal points around campus; nylon netting was purchased from a local supplier and urethane foam was collected from a housing dumpsite, all of which were then cut into smaller pieces. 100 g of mixed plastic waste comprising PET (49.0 g), polyamide (35.8 g), and polyurethane (15.3 g) were added in NMP (120 g) at 180 °C. 4 waste bottle caps and PET bottle packaging labels (1.0 g) were also added as inert participants. Mixed plastic waste was then depolymerized with glycerol (10.0 g) and [EMIM] Cl (1.2 g) as catalyst for 18 h at 180 °C. A layer of polyolefins was observed on the top of the reaction mixture due to phase separation, which was removed via decanting. PET, polyamide, and polyurethane plastic products were observed to be completely solubilized. Waste plastic oligomers were then precipitated in deionized water (500 mL) with stirring to obtain a dark red powder. Precipitate was removed via vacuum filtration and placed into 500 mL of deionized water with stirring for another washing cycle. This cycle was repeated twice. The final removal of the precipitate was through vacuum filtration, and the precipitate was then dried in a convection oven at 80 °C to afford recovered waste mix plastic (RMP), yield of polymeric material recovery was calculated against the initial mass of mixed plastics (100 g): 80.0 g, yield of recovery: 80%.

### RMP vitrimer (vRMP) film fabrication

20 g of RMP powder was dry mixed with 1.37 g Zn(acac)_2_ and fed into Thermo Scientific HAAKE MiniLab II twin screw extruder, 220 °C at 80 rpm. The extruded product was then fed back into the machine and extruded again at the same settings for an additional two times. The extruded sample was then heat-treated in a convection oven at 250 °C for 2 h. The resultant was then hot pressed at 250 °C for 30 min to produce a mixed plastic oligomeric derived co-polymer film for DSC testing.

### Fine glass powder

A glass bottle was shattered with a hammer and ground in a blender to afford coarse waste glass powder. Coarse glass powder was then sieved with a mesh size of 150 microns to afford fine waste glass powder with a diameter of less than or equal to 150 microns. The fine glass powder is then used as filler for subsequent composite fabrication with RMP.

### vRMP glass composite fabrication

20 g of RMP, 80 g of fine glass powder, and 1.37 g of Zn(acac)_2_ were dry mixed and fed into the Thermo Scientific HAAKE MiniLab II twin screw extruder. The powder mixture was melted and extruded at 220 °C at 80 rpm. The extruded product was then fed back into the machine and extruded again at the same settings for an additional two times. The extruded sample was hot pressed at 250 °C and 20 MPa for 5 min in a (12 cm (length) × 12 cm (width) × 0.1 cm (thickness)) square frame and left to cool to room ambient temperature to achieve a square tile. The resultant tile was then thermally cured in a convection oven at 250 °C for 2 h to afford the RMP composite tile.

### Fourier-transform infrared spectroscopy (FTIR)

Fourier-transform infrared spectroscopy was carried out using Perkin–Elmer Frontier equipment in transmission mode. Data collected were averaged over 16 scans from the 400–4000 cm^−1^ wavenumber range at a resolution of 4 cm^−1^. Sample powder was ground and mixed with dry potassium bromide powder then cold-pressed into pellets for analysis.

### Rheology

#### RPET and PET isothermal oscillation

A TA instrument, DHR-3, was used to perform isothermal time sweep oscillation rheology using an 8 mm diameter steel parallel plate, within a environmental test chamber accessory. Isothermal oscillation rheology was done on RPET 1:1, RPET 5:3, and RPET 5:1 powder samples mixed with 5 mol% zinc acetylacetonate and on neat PET as the control. Samples were melted and placed between parallel plates, at a 550 µm gap, under a constant mechanical oscillation frequency of 1 Hz and 1% strain. RPET samples were loaded at 250 °C to achieve a melt and the system was then allowed to equilibrate to 220 °C for isothermal oscillation. Neat PET was loaded at 280 °C to achieve a melt and the system was then allowed to equilibrate to 260 °C for isothermal oscillation. Isothermal oscillation rheology was conducted on all RPET samples at 220 °C, and PET samples at 260 °C, for 2 h.

#### vRPET and PET small angle oscillation

A TA instrument DHR-3 was used to perform small angle oscillation rheology using an 8 mm diameter steel parallel plate, within a environmental test chamber accessory. vRPET 1:1, vRPET 5:3, vRPET 5:1, and PET were cold pressed into a cylindrical pellet with a diameter of 10 mm and ~1.5 mm thick. vRPET samples were then sandwiched between parallel plates at 180 °C while the PET sample was loaded at 230 °C. Isothermal frequency sweeps from 0.01 to 500 rad/s were done at a temperature range from 180 to 270 °C for vRPET samples, and a temperature range of 230–275 °C for the PET sample, with a strain of 1% which is in the linear viscoelastic regime.

#### Isothermal rheology on RMP

A TA instrument, DHR-3, was used to perform isothermal time sweep oscillation rheology using an 8 mm diameter steel parallel plate, within a environmental test chamber accessory. RMP was dry mixed with the same wt% of zinc (from zinc acetylacetonate) as RPET samples. A powder sample was placed between the parallel plates, at a 500 µm gap, with the application of a constant mechanical oscillation frequency of 1 Hz and 1% strain. The RMP sample was then observed from room temperature and equilibrated to 220 °C for 2 h.

### Dynamic mechanical analysis (DMA)

The DMA method was adapted from work done by Zhou et al.^35^. Hot-pressed vRPET films were cut into (ca. 20.4 mm (length) × 9.0 mm (width) × 0.2 mm (thickness)) rectangular strips and analyzed using a DMA Q800 (TA Instruments) using a film tension set up. A temperature sweep from room temperature (~28 °C) to 270 °C was done at a rate of 3 °C/min at a frequency of 1 Hz. A preload force of 0.01 N, an amplitude of 10 µm and a force track of 125% was implemented.

#### DMA Heat Cool Cycle for vRPET films

Heating and cooling cycles of DMA were performed on a vRPET. vRPET thin film was cut into a cut into a (ca. 18.9 mm (length) × 9.6 mm (width) × 0.10 mm (thickness)) rectangular strip and measured on a DMA Q800 (TA Instruments) with a film tension set up. A temperature ramp from room temperature (~28 °C) at 3 °C/min to 270 °C, followed by an uncontrolled cooling to 40 °C and a final heating ramp of 3 °C/min to 270 °C was performed at a frequency of 1 Hz. A preload force of 0.01 N, an amplitude of 10 µm, and a force track of 125% was implemented.

### X-ray diffraction (XRD) analysis

Wide-angle x-ray diffraction was performed using a Bruker D8 Advance diffractometer using Cu-Kα radiation, wavelength of 1.5418 Å, at 40 kV and 40 mA. Scans were performed at 30 rpm and with a scan rate of 0.0125°/s (2*θ*; 0.025°/s) in a 2*θ* range of 10–80° with an amorphous silicon substrate. The analyses were performed on hot-pressed samples of vRPET 1:1 and PET.

### Differential scanning calorimetry (DSC)

TA Instruments Q10 DSC was used to study the thermal behavior of all samples. DSC analysis was carried out on samples encased in aluminum hermetic pans. Samples were subjected to a flow rate of 50 cc/min of flowing nitrogen gas, a heating ramp of 10 °C/min from 20 to 270 °C, and a cooling ramp of 10 °C/min to 10 °C for virgin PET and RPET samples.

#### RMP powder and film DSC

DSC analysis for RMP powder and film was carried out on samples encased in aluminum hermetic pans. RMP powder and film samples were subjected to, under N_2_ atmosphere, a heating ramp of 10 °C/min from 20 to 320 °C and 300 °C, respectively, and a cooling ramp of 10 °C/min to 10 °C for both samples.

### Gel permeation chromatography (GPC)

GPC method was adapted from an instructional article provided by Agilent^60^. GPC was carried out using the Agilent 1260 Infinity system through Agilent PLgel Sum Mixed-C followed by Agilent PLgel Sum Mixed-E columns. The system was calibrated with polystyrene standards ranging from 162 Da to 45.12 kDa. 10 mg of the sample was dissolved in 0.2 mL of Hexafluoro-2-propanol (HFIP). A further 4.8 mL of chloroform was added resulting in a total concentration of 2 mg/mL. Sample solution was filtered through a 0.2 μm PTFE filter. The sample was eluted with chloroform at a flow rate of 1 mg/mL.

### Thermogravimetric analysis (TGA)

To ascertain the amount of glass present in the RMP composite, TGA was performed using the TA Instruments Q500 thermogravimetric analyzer (TGA) on a sample of RMP. 21.6 mg of RMP composite, under 50 cc/min of flowing nitrogen gas, was placed at an isothermal temperature of 80 °C for 10 min to dry the sample. The temperature was then raised to 800 °C at a ramp rate of 10 °C/min.

### Tensile mechanical testing

Six dog-bone-shaped specimens were cut from the bulk vRPET films to produce a uniform profile of samples with a long central of (*l* × *w* × *t*) 10 mm × 3 mm × ~0.085 mm to control the localization and development of fracture at the middle of the specimen. Tensile testing was performed by an Instron 5567 Electric Universal Testing Machine at a strain rate of 5 mm/min.

### 3-Point bending test

3-point bend testing was performed by an Instron 5567 Electric Universal Testing Machine with a support span of 30 mm and a loading span of 10 min. 2 RMP glass composite was prepared in accordance with the method mentioned above. From the 2 tiles, 21 smaller tiles were cut and ground to dimensions of approx. (*l* × *w* × *t*) (50 × 13 × 1.4) mm with smoothened sides. 3-point bending test was then performed at a strain rate of 10 mm/min.

#### RMP glass composite recyclability experiment

RMP glass composite, after initial 3-point testing on pristine sample set, was manually broken into small shards and placed into a (12 cm (length) × 12 cm (width) × 0.1 cm (thickness)) square frame and hot pressed at 250 °C, 26 MPa for 5 minutes. Additional RMP composite shards, subjected to the same thermal conditions and hot-press reprocess cycles, were added to fill any gaps in the square frame and hot pressed at the same conditions for an additional two more times, affording a total time of 15 minutes, to produce a whole square tile. The tile was then cut and grinded to afford 7 smaller samples of approx. (*l* × *w* × *t*) (50 × 13 × 1.4) mm with smoothened sides. Through this process, it would be considered 1 cycle of reprocessing. RMP glass composite would be subjected to 5 cycles of reprocessing for 3-point testing at a strain rate of 10 mm/min for each sample.

## Supplementary information

Supplementary Information

## Data Availability

The authors declare that the findings reported in this study are supported by the data published in the paper and its Supplementary Information. In the event raw data files are required in other formats, there are obtainable, upon reasonable request, from the corresponding author.
