# Supplementary material for: A facile alternative strategy of upcycling mixed plastic waste into vitrimers
Source: Commun Chem. 2023 Jul 27;6:158. doi: 10.1038/s42004-023-00949-8 (PMC10374618; doi:10.1038/s42004-023-00949-8)
Supplement: Supplementary file 1 — Supplementary Information [file 42004_2023_949_MOESM1_ESM.pdf]

Supplementary Information

A Facile Alternative Strategy of Upcycling Mixed Plastic Waste into Vitrimers

*Kok Wei Joseph Ng [1, ‡], Jacob Song Kiat Lim [2, ‡], Nupur Gupta [1], Bing Xue Dong [1], Chun-Po Hu [3], Jingdan Hu [1], Xiao Matthew Hu [1,2,3,]**

[1] *School of Material Science and Engineering, Nanyang Technological University, Nanyang Avenue, 639798 Singapore, Singapore*

[2] Temasek Laboratories, Nanyang Technological University, 50 Nanyang Drive, 637553 Singapore

[3] *Nanyang Environment and Water Research Institute, Nanyang Technological University, 637141 Singapore*

‡These authors contributed equally

*Corresponding Author, Email: Asxhu@ntu.edu.sg

KEYWORDS

Upcycling, Mixed plastic waste, Glycolysis, Controlled partial depolymerization, Branched oligomer, Vitrimers

Table S1: Summary of molecular weights of sample after size exclusion chromatography calculated and reported using Agilent 1260 Infinity system.

**Sample**	**M_n (Da)**	**M_w (Da)**	**PDI**
PET	32060	54802	1.709
RPET 1:1	694	1220	1.758
RPET 5:3	970	1871	1.929
RPET 5:1	1372	3239	2.361

Table S2: Reagent formulation to afford vRPET samples.

**RPET Feedstock**	**RPET Mass (g)**	**Amt of RPET (mol)**	**$Zn(acac)_2$ mol%**	**$Zn(acac)_2$ wt%**	**Amt of $Zn(acac)_2$ (mol)**	**Mass of Zn Cat. (g)**
RPET 1:1	7	0.03646	5.00%	6.42%	0.0018	0.4805
RPET 5:3	10	0.05208	5.00%	6.42%	0.0026	0.6865
RPET 5:1	10	0.05208	5.00%	6.42%	0.0026	0.6865

Gel permeation chromatography (GPC)

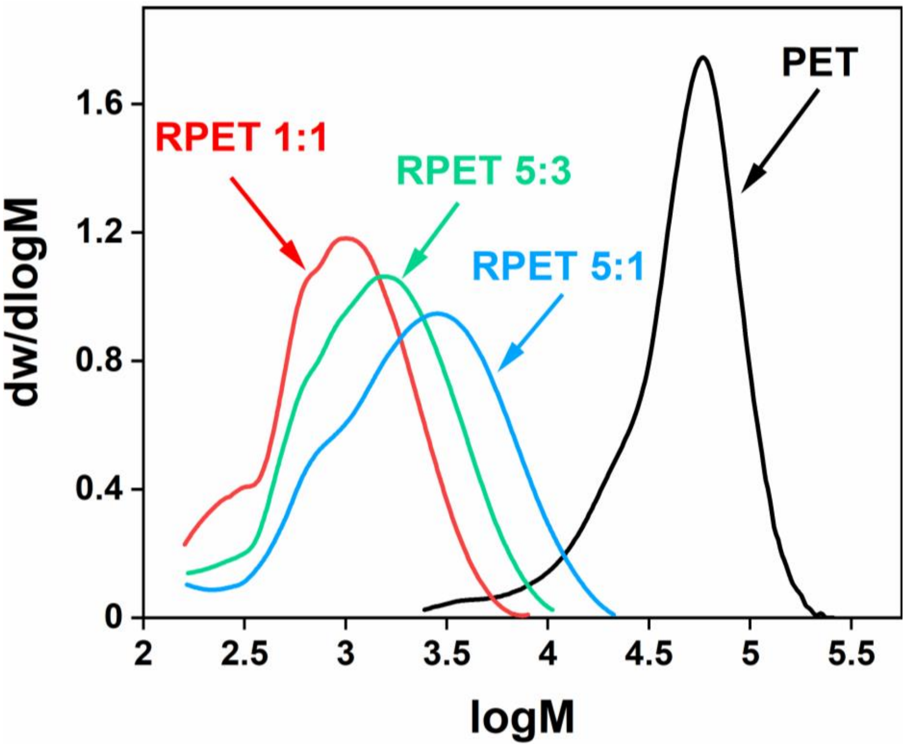

Figure S1: Molecular weight distribution of PET (black) and RPET samples (RPET 1:1 (red), RPET 5:3 (green) and RPET 5:1 (blue)).

Supplementary Note 1:

Proposed Kinetics of Crosslinking through Transesterification Interactions

$$Ester^1 + (-OH)^1 \leftrightarrow Ester^2 + (-OH)^2 ------ (1)$$

Assuming that the reactivities of all hydroxyl species are similar, and the system is a well-mixed and homogenous, the general transesterification reaction is given in equation (1). It is an equilibrium reaction between esters and hydroxyl groups, where ester species would exchange its hydroxyl unit with another hydroxyl unit to form a new ester.

$$Rate = k[Ester][-OH] ------ (2)$$

Hence, the rate of the exchange reaction (both forward and backwards) can be given by rate equation (2), where k is the rate constant. If k and [Ester] are constants, the rate of the forwards and backwards reaction would be proportional to the concentration of hydroxyl groups in the system. (i.e. the higher the amount hydroxyl groups, the faster the exchange back and forth would be.). Therefore, at higher magnitudes of [-OH] it increases the possibilities of lower boiling point hydroxyl species to be removed from the system at higher temperatures, thus driving the equilibrium reaction to the right. Glycerol, having a high boiling point (290 °C), is unlikely to be removed from the system by vaporization at the reaction temperature. This allows for glycerol, a trifunctional polyol, to be inserted into the polymer chain, substituting out hydroxyl containing species, which ultimately crosslinks the polymer. The faster the reversable exchange reaction, the higher the probability of glycerol substituting out hydroxyl containing species which would lead to faster crosslinking.

RPET 1:1 Molded sample

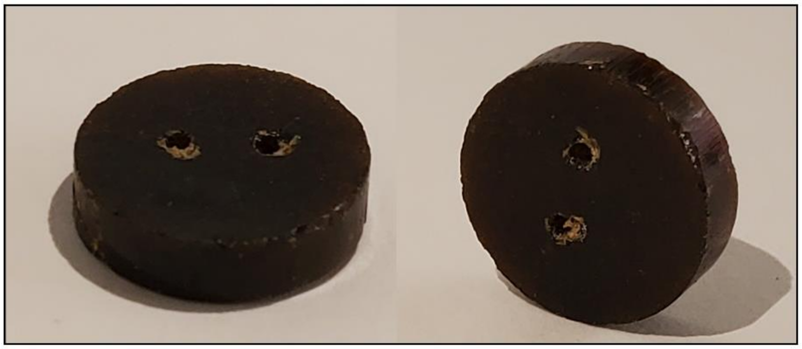

Figure S2: vRPET 1:1 molded into a thicker “button-like” disk.

DMA Analysis

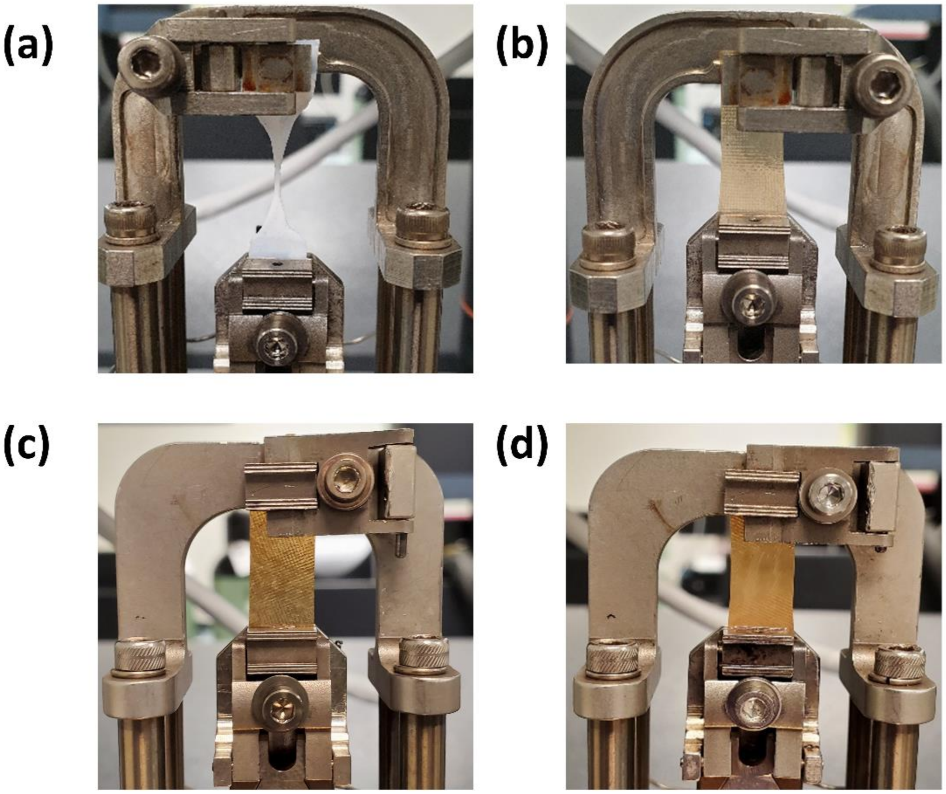

Figure S3: vRPET and PET films post DMA test. (a) Virgin PET film yielded at temperatures ~260 °C (b) vRPET 1:1 (c) vRPET 5:3 (d) vRPET 5:1 films did not yield after DMA testing at temperatures above 250 °C.

DMA Heat Cool Cycle for vRPET films

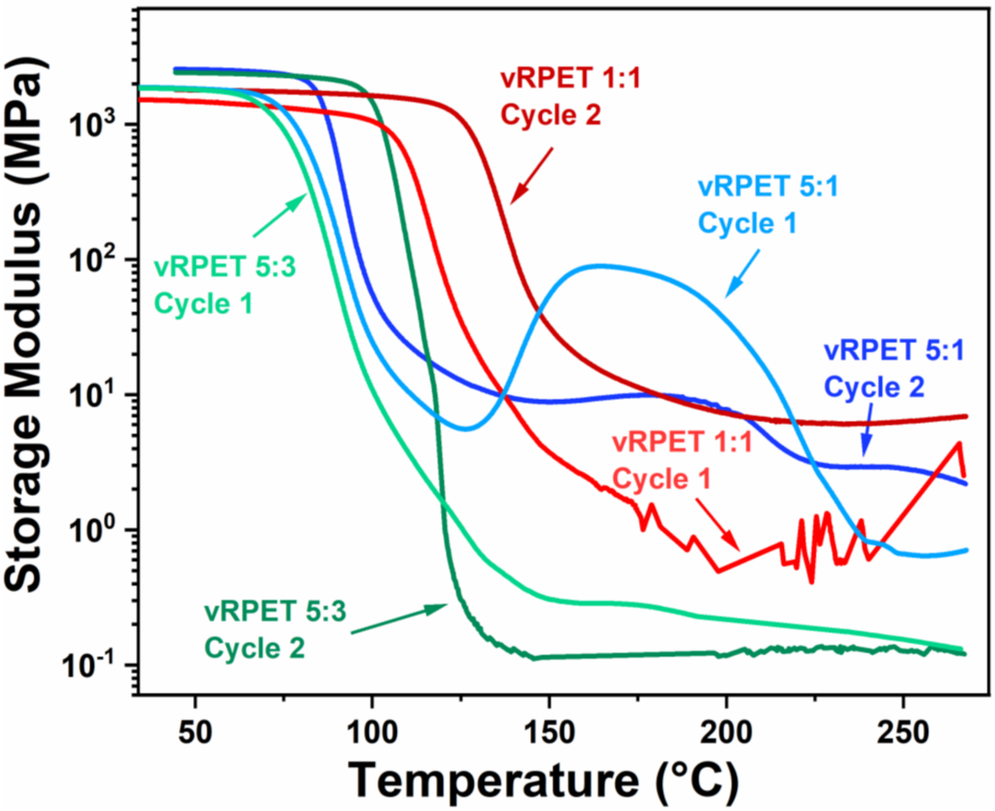

Figure S4: DMA storage modulus of vRPET films after two cycles of heating. First cycle was done from room temperature to 270 °C (lighter colors; vRPET 1:1 (red), vRPET 5:1 (blue) and vRPET 5:3 (green)) followed by uncontrolled cooling to 40 °C (blue) then reheating to 270 °C (darker colors; vRPET 1:1 (red), vRPET 5:1 (blue) and vRPET 5:3 (green)).

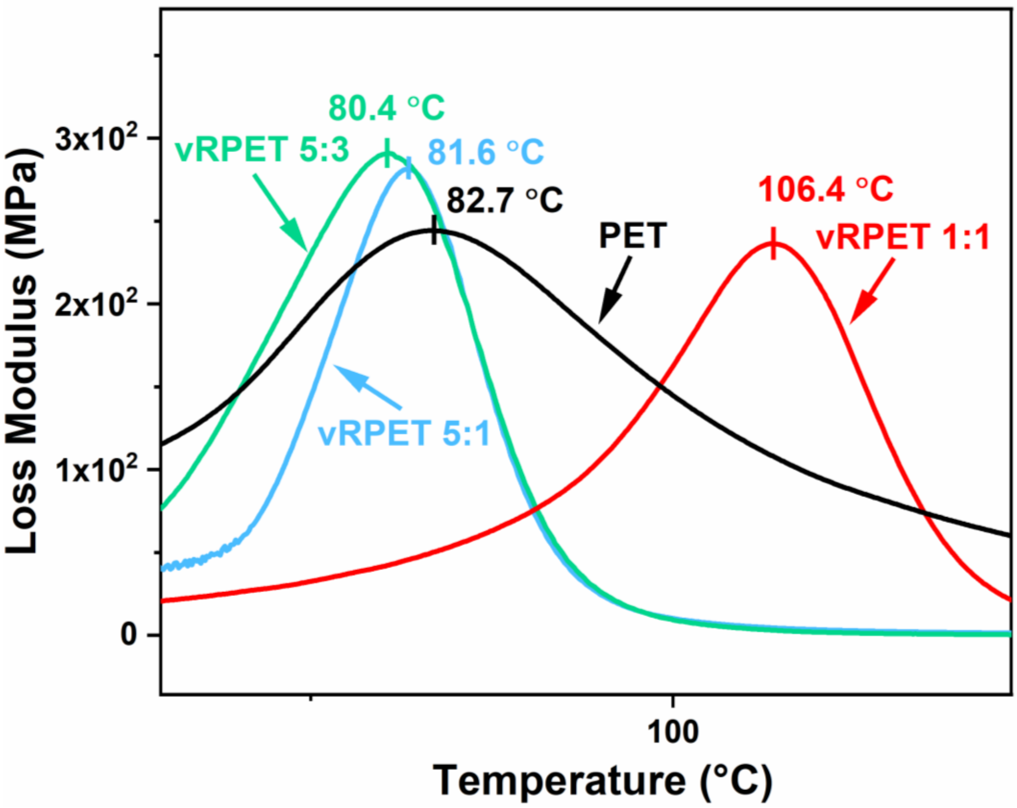

Figure S5: DMA loss modulus (E”) of neat PET (black), vRPET 5:1 (blue), vRPET 5:3 (green) and vRPET 1:1 (red). Glass transition (T_g) of each sample is calculated by the maxima of the E” curves.

Tensile Mechanical Testing

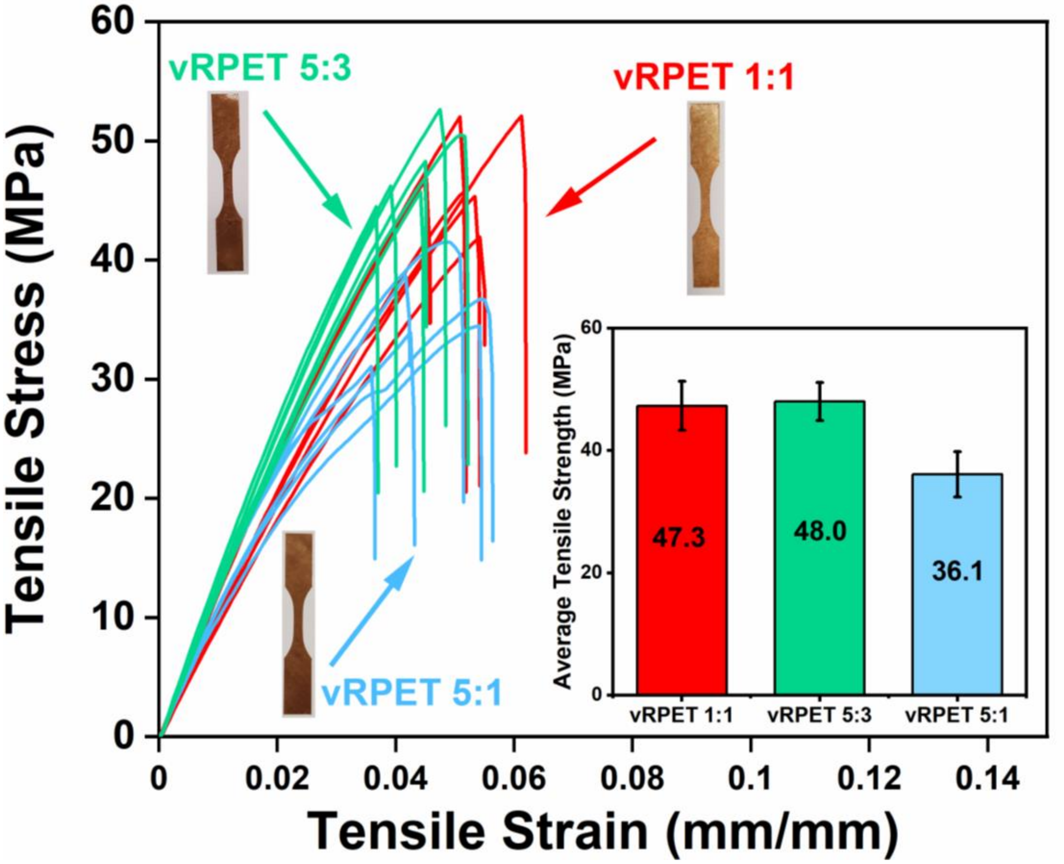

Figure S6: Tensile stress-stain curves of six sample films of vRPET 1:1 (red), vRPET 5:3 (green) and vRPET 5:1 (blue) respectively. Bar-graph insert: Average tensile strength of vRPET samples with error bars from sample variations.

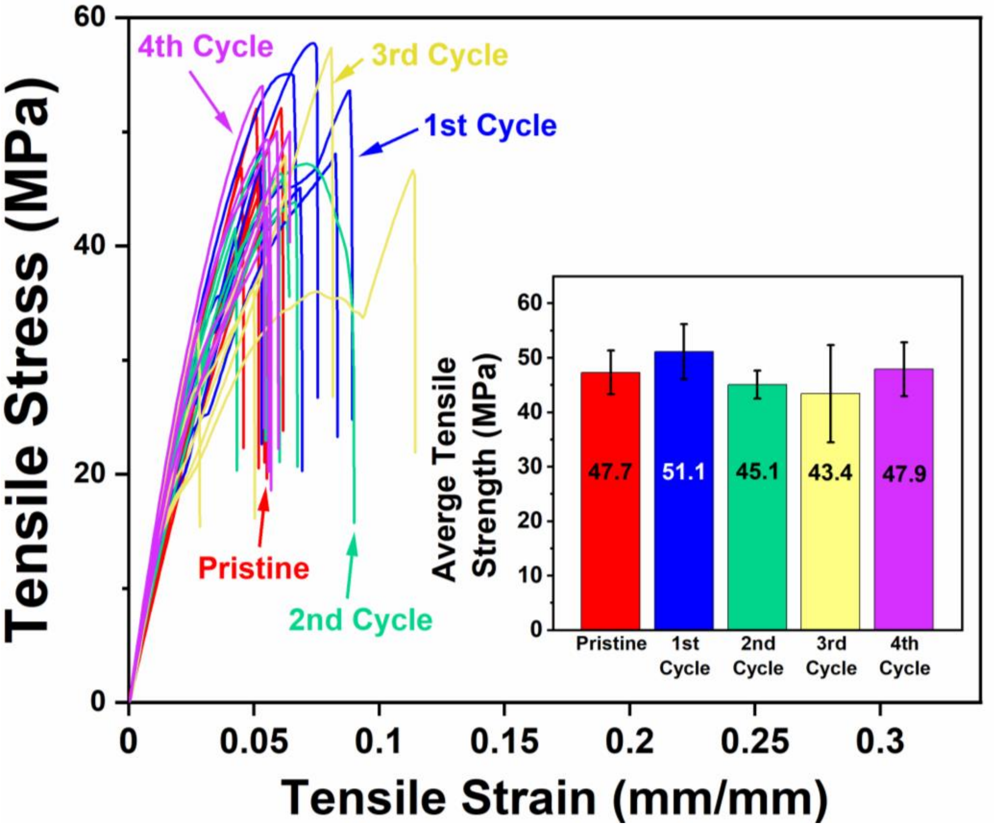

Figure S7: Tensile stress-stain curves of six sample films of vRPET 1:1 film (red) reprocessed for 1 cycle (blue), 2 cycles (green), 3 cycles (yellow) and 4 cycles (purple). Bar-graph insert: Average tensile strength of vRPET 1:1 and reprocessed vRPET samples with error bars from sample variations.

Thermogravimetric analysis (TGA)

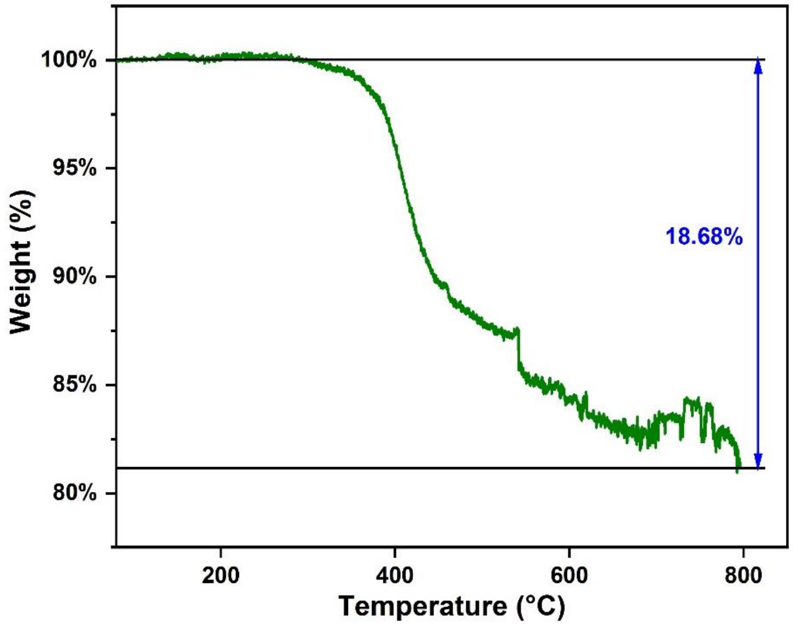

Figure S8: TGA curve of RMP glass composite to determine amount of glass present in extruded sample.

Rheological Experiments

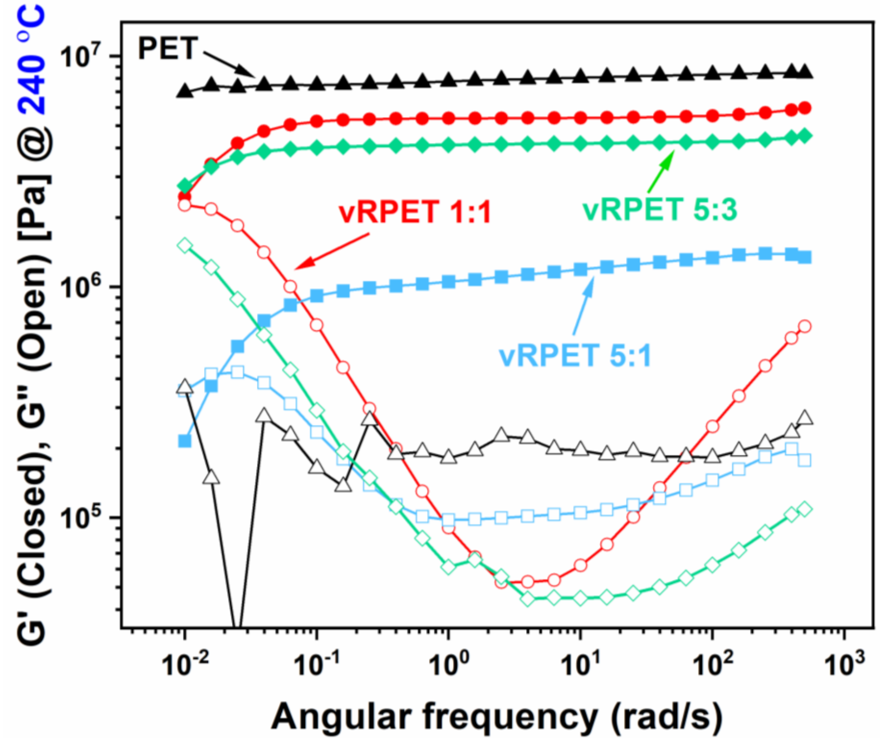

Figure S9: Isothermal small angle oscillation of neat PET, vRPET samples at 240 °C.

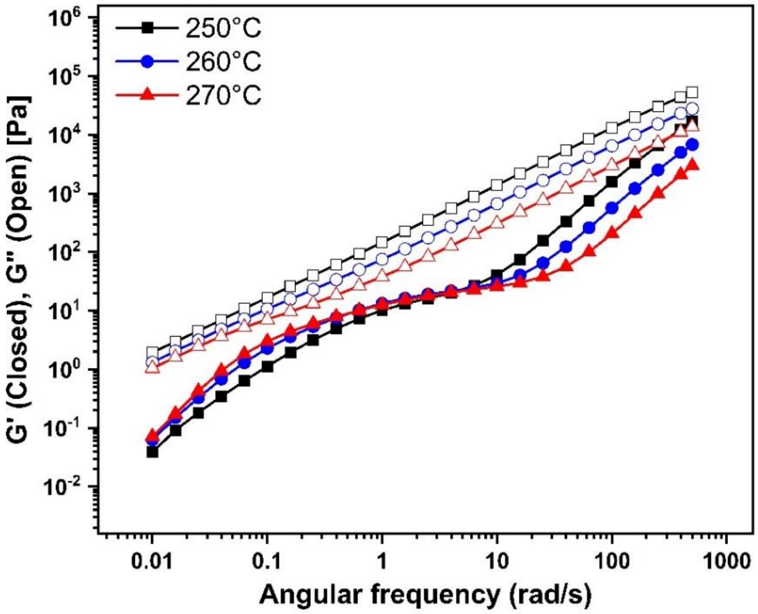

Figure S10: Small angle oscillation rheology on PET melt at 250 °C (black), 260 °C (blue) and 270 °C (red). Open and close symbols represent G" and G' of the sample respectively.

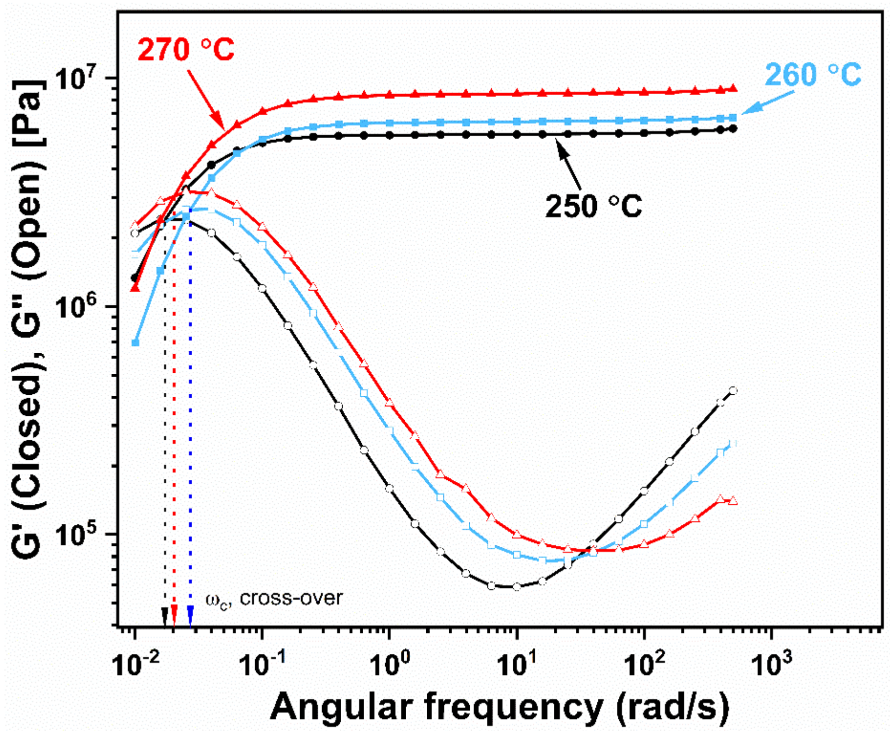

Figure S11: Small angle oscillation rheology on vRPET 1:1 at 250 °C (black), 260 °C (blue) and 270 °C (red). Open and close symbols represent G” and G’ of the sample respectively.

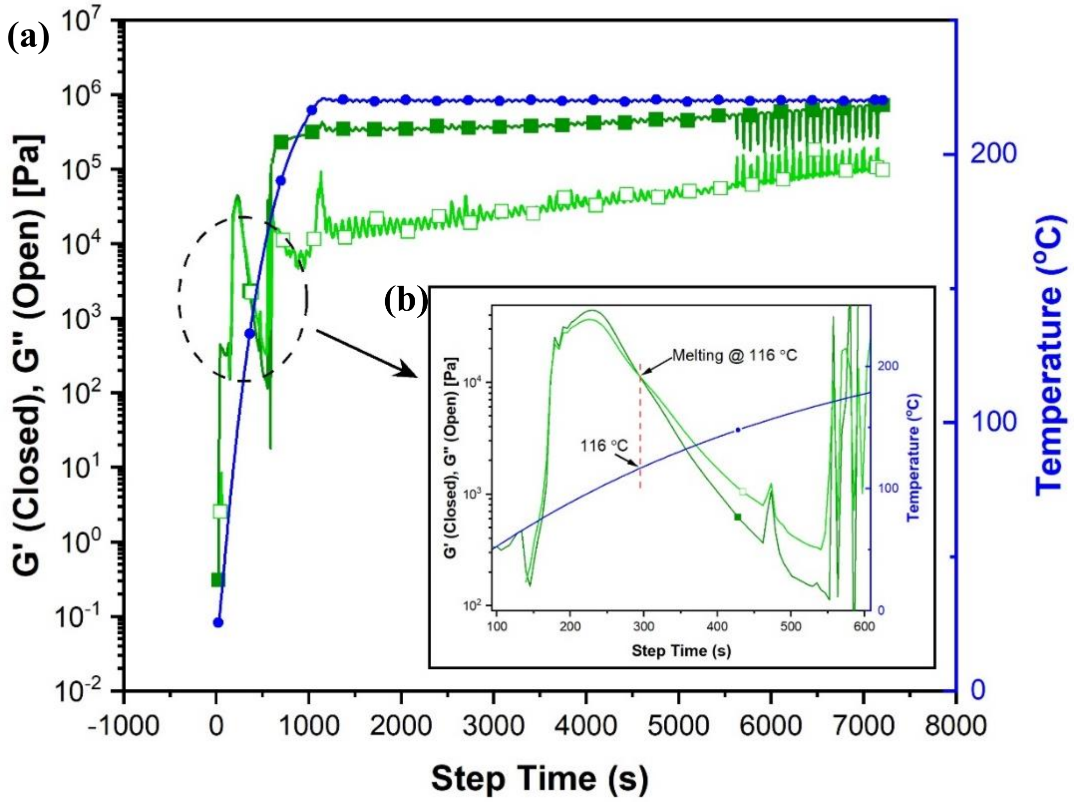

Figure S12: (a) Isothermal oscillation rheology of RMP with catalytic amount of $Zn(acac)_2$. Sample was loaded in at room temperature to observe melt modulus behavior (green). Temperature (blue) quicky increase to 220 °C which maintained for 2 hours. (b) Zoom-in of area of the graph within the dashed circle. RMP powder melts and behaved as an oligomer melt at 116 °C.

X-ray Diffraction (XRD) Analysis

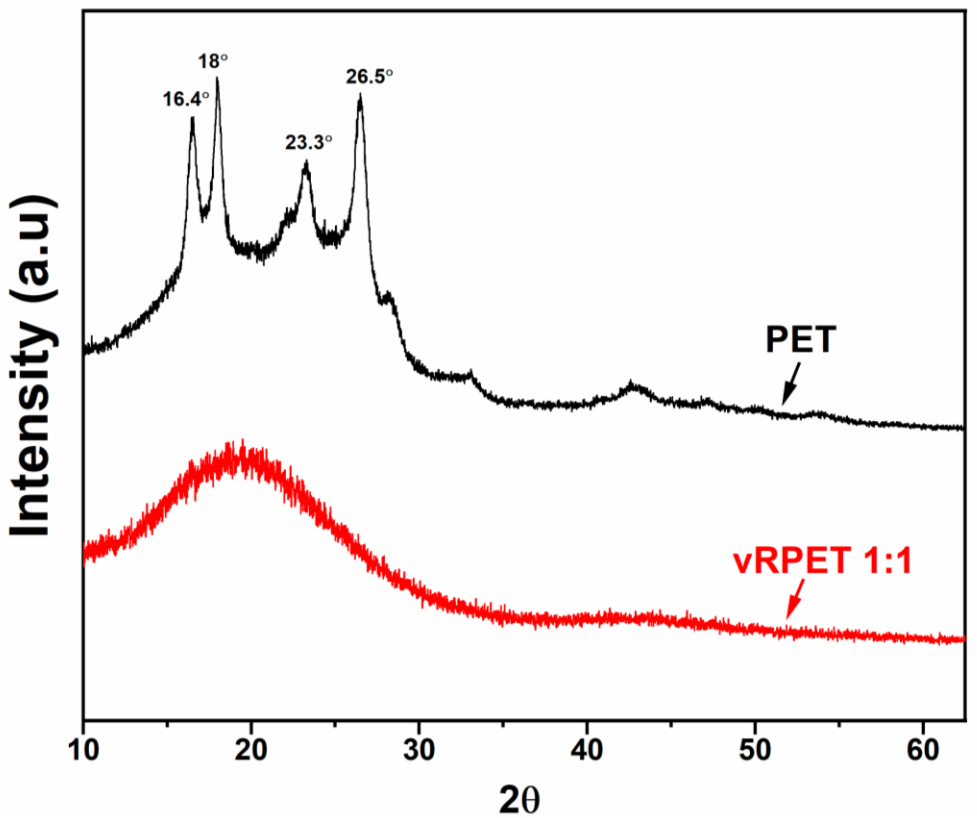

Figure S13: XRD diffractogram of PET and vRPET 1:1 film.

Fourier transform infrared spectroscopy (FTIR)

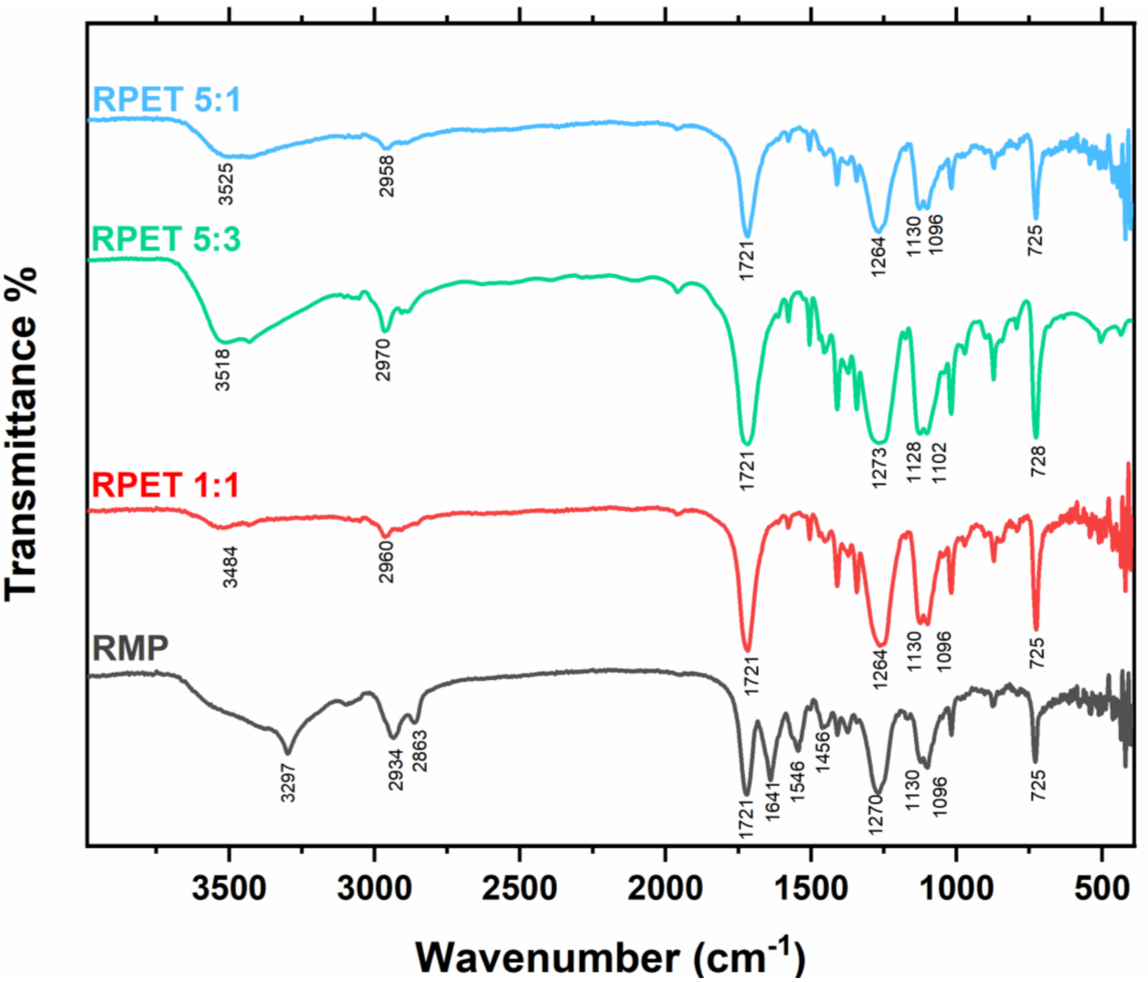

Figure S14: FTIR spectra of RPET 5:1 (blue), RPET 5:3 (green) and RPET 1:1 (red) and RMP (grey) powders for wavenumbers 4000 cm^{-1} to 400 cm^{-1}.

Differential scanning calorimetry (DSC)

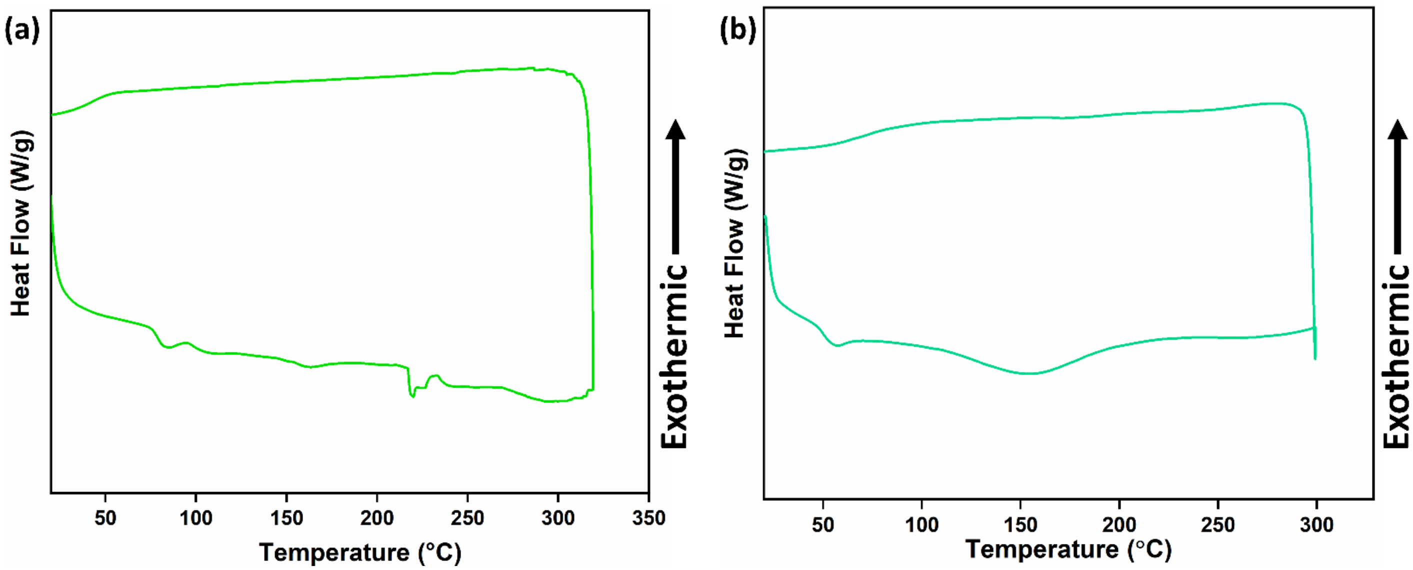

Figure S15: (a) DSC curve of RMP oligomeric powder. (b) DSC curve of polymeric RMP film after heat treatment.

Scanning Electron Microscope Imaging (SEM)

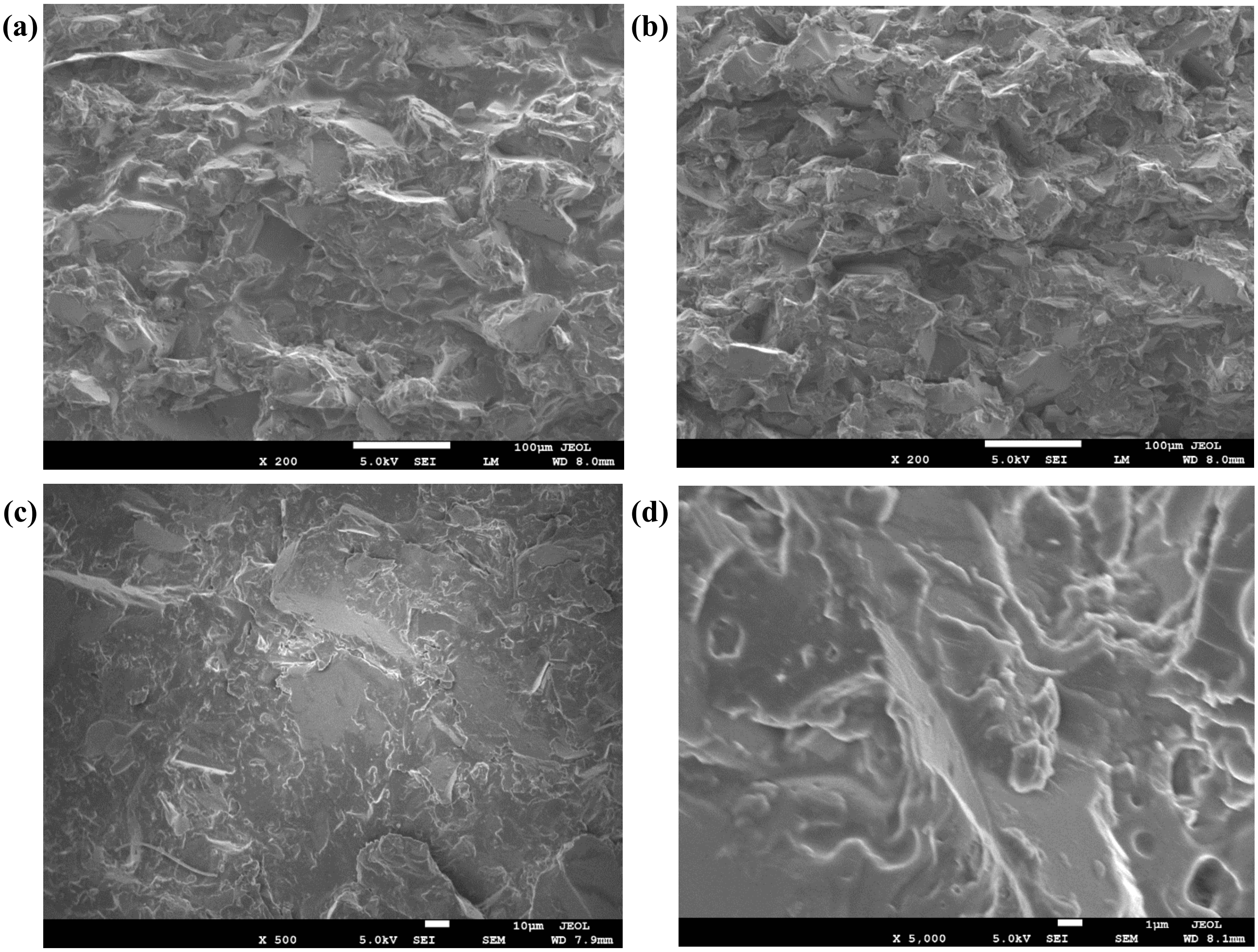

Figure S16: Visualization of the composite cross-section was attained using a FESEM JEOL JSM-7800F PRIME Scanning electron microscope. SEM imagining of the cross section of extruded RMP composite tile. (a-b) Magnification at 100 μm. (c) Magnification at 10 μm. (d) Magnification at 1 μm.

3-Point Flexure Bend Mechanical Testing

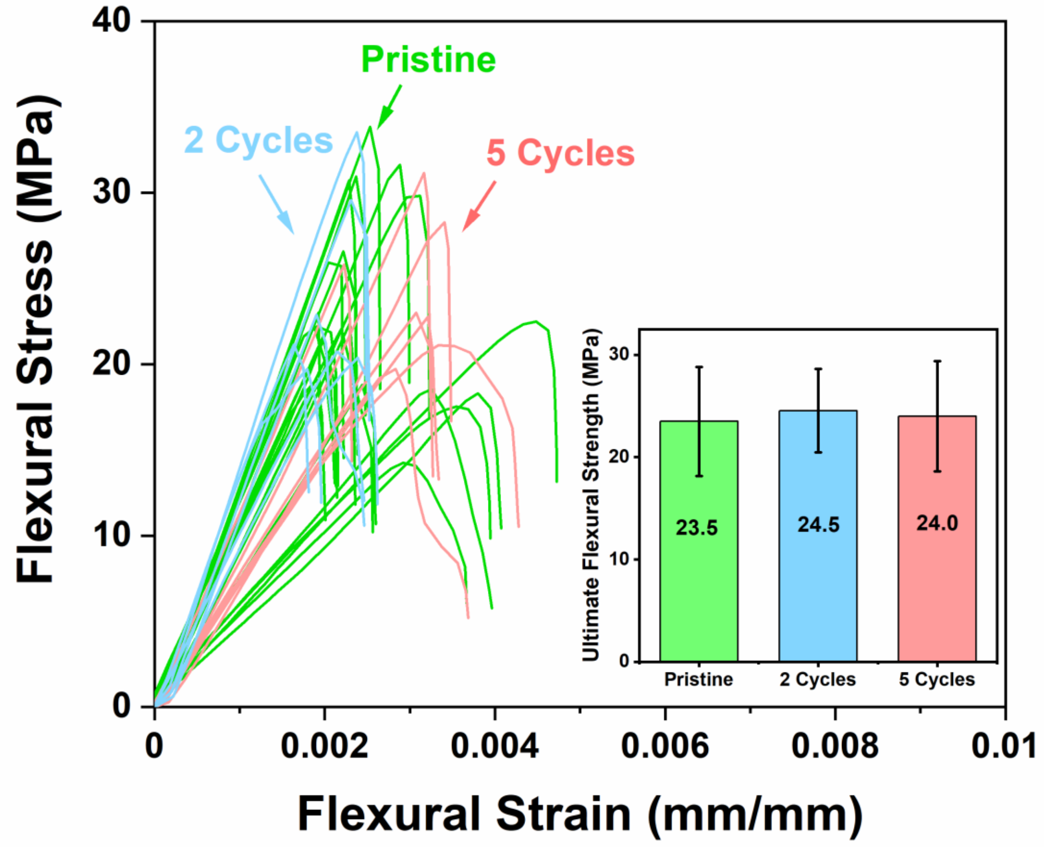

Figure S17: Flexure stress-stain curves of 21 samples of pristine (green), 7 samples of "2 cycles reprocessed" (blue) and 7 samples of "5 cycles reprocessed" (red) RMP composite comprising of 80wt% of glass. Bar-graph insert: Ultimate flexural strength of RMP glass composite samples with error bars.
